# Survival and neurological outcome in patients treated with extracorporeal membrane oxygenation and therapeutic hypothermia: an updated systematic review and meta-analysis

**DOI:** 10.3389/fmed.2026.1882223

**Published:** 2026-07-10

**Authors:** Bin Miao, Pengfei Cheng, Jianfeng Xu, Bei Liu, Qianmi Wang

**Affiliations:** 1 Department of Nursing, The Second Affiliated Hospital of Zhejiang University School of Medicine (SAHZU), Hangzhou, Zhejiang, China; 2 Faculty of Medicine, Macau University of Science and Technology, Macau, Macao SAR, China

**Keywords:** cardiac arrest, extracorporeal cardiopulmonary resuscitation, meta-analysis, neurological outcome, targeted temperature management, therapeutic hypothermia

## Abstract

**Objective:**

To assess the impact of therapeutic hypothermia on survival, neurological outcome, and complications in adult patients with cardiac arrest undergoing extracorporeal cardiopulmonary resuscitation (ECPR).

**Methods:**

A systematic search of PubMed, Embase, Web of Science, the Cochrane Library, and major Chinese databases was conducted from their inception to March 31, 2026. Randomized controlled trials (RCTs) and non-randomized studies of interventions comparing therapeutic hypothermia with normothermia or fever prevention alone in adult patients undergoing ECPR were included. The primary outcomes were survival and favorable neurological outcome. The secondary outcome was the incidence of complications. A random-effects model was used to calculate pooled risk ratios (RRs) and their 95% confidence intervals (CIs), with results stratified by study design.

**Results:**

A total of 31 studies involving 6,184 ECPR patients were included, of which only 3 were RCTs. Pooled randomized trials did not demonstrate a significant benefit of therapeutic hypothermia for either survival to hospital discharge (RR = 1.30, 95% CI: 0.70–2.40, *P* = 0.41) or favorable neurological outcome at discharge (RR = 1.80, 95% CI: 0.86–3.77, *P* = 0.12), and no benefit was demonstrated at 1, 3, or 6 months. In contrast, observational studies showed that therapeutic hypothermia was associated with better survival (RR = 1.41, 95% CI: 1.14–1.74, *P* = 0.002) and more favorable neurological outcomes (RR = 1.61, 95% CI: 1.21–2.14, *P* = 0.001), though these were based on crude, unadjusted data with substantial heterogeneity. Among these observational studies, the associations appeared confined to a moderate target temperature (32.0–34.0°°C; survival RR = 1.73; neurological RR = 2.27), with no significant association at mild temperatures (34.0–36.0 °C). Therapeutic hypothermia was not associated with an increased risk of major complications in ECPR patients.

**Conclusions:**

Pooled randomized evidence did not demonstrate better survival or more favorable neurological outcomes with therapeutic hypothermia in ECPR patients; more favorable outcomes were confined to crude observational data at high risk of bias. With certainty of evidence rated low to very low, adequately powered randomized trials are needed before firm conclusions can be drawn.

**Systematic review registration:**

https://www.crd.york.ac.uk/prospero/, identifier CRD42023435353.

## 1 Introduction

Cardiac arrest remains a leading cause of death worldwide, and despite decades of advances in resuscitation science, survival to discharge and favorable neurological recovery remain persistently poor across regions (1, 2). Survival to hospital discharge after out-of-hospital cardiac arrest (OHCA) is approximately 10% in the United States (2) and 7.5% in Europe (3), and as low as 1.2–2.8% in China (3, 4); even among in-hospital cardiac arrest, fewer than one in four patients survive, and a large proportion of survivors are left with lasting neurological impairment (2, 4, 5). These figures underscore that cardiac arrest remains a formidable clinical challenge and highlight the need for more effective therapeutic strategies.

To overcome the limitations of conventional cardiopulmonary resuscitation (CPR) in refractory cardiac arrest, extracorporeal cardiopulmonary resuscitation (ECPR) has emerged as an increasingly used life-sustaining technology (6). By rerouting blood through an extracorporeal membrane oxygenation (ECMO) circuit, ECPR temporarily assumes cardiopulmonary function, creating a time window to address the underlying cause while maintaining organ perfusion and limiting neurological injury (7). Registry data from the Extracorporeal Life Support Organization (ELSO) indicate that approximately 30% of ECPR patients survive to discharge or transfer, of whom about 17% achieve a favorable neurological status (8). Although ECPR has broadened treatment options for refractory cardiac arrest, these outcomes still fall well short of ideal, motivating investigation of adjunctive strategies—including therapeutic hypothermia—that may further improve prognosis (7, 9).

Therapeutic hypothermia has long been considered a component of post-resuscitation care, with neuroprotective effects attributed to reductions in cerebral metabolic demand and attenuation of ischemia–reperfusion injury (10–13). This consensus, however, has been substantially challenged. The landmark Targeted Temperature Management 2 (TTM-2) trial found that targeting 33°C, compared with fever prevention alone, did not improve 6-month survival or neurological outcome and was associated with more arrhythmias (14), and a 2022 systematic review reported no benefit in non-shockable arrest (15). Consequently, the European Resuscitation Council and the European Society of Intensive Care Medicine revised their recommendations from active hypothermia toward active fever prevention (16), while the American Heart Association adopted a broader target range of 32–37.5°C (17). As a result, deep hypothermia is no longer a uniform or routine standard of post-arrest care, and contemporary temperature management remains heterogeneous across centers and countries.

In contrast to the TTM-2 trial, a large-scale randomized controlled trial (RCT) by Lascarrou et al. (18) reported a significant neurological benefit with moderate therapeutic hypothermia, highlighting the persistent uncertainty in this field. This discrepancy is particularly relevant to the ECPR subgroup, because the pathophysiology of refractory cardiac arrest managed with extracorporeal support differs markedly from that of conventional cardiac arrest. Consequently, direct extrapolation of findings from the general cardiac arrest population to ECPR patients is widely regarded as methodologically unreliable (19). The evidence specific to ECPR, however, remains limited and conflicting. A 2020 meta-analysis by Chen et al. (20), synthesizing data from multiple observational studies, found that therapeutic hypothermia was associated with higher survival rates and more frequent favorable neurological outcomes in this population. Reflecting this uncertain evidence base, a joint statement from the European Society of Emergency Medicine (EuSEM) and the European Society of Anesthesiology and Intensive Care (ESAIC) concluded that currently available evidence is insufficient to justify abandoning the intervention, while explicitly calling for further rigorous investigation to delineate the specific patient subgroups most likely to derive clinical benefit (21). The optimal temperature strategy in ECPR therefore remains genuinely unresolved.

Given these inconsistencies among large-scale randomized trials, prior systematic reviews, and international guidelines, the role of therapeutic hypothermia in ECPR patients remains controversial (7). Moreover, the rapid global expansion of ECPR since the COVID-19 pandemic has generated a substantial volume of new clinical data (6), the inclusion of which may increase statistical power and help resolve this uncertainty. We therefore conducted an updated systematic review and meta-analysis to evaluate the association between therapeutic hypothermia and survival, favorable neurological outcomes, and major complications in adults receiving ECPR, in order to inform current clinical practice.

## 2 Methods

### 2.1 Protocol and registration

This systematic review and meta-analysis was reported in accordance with the Preferred Reporting Items for Systematic Reviews and Meta-Analyses (PRISMA) 2020 statement (22). The study protocol was prospectively registered in the International Prospective Register of Systematic Reviews (PROSPERO) (registration number: CRD42023435353), and its detailed methodology has been previously published as a standalone paper in *BMJ Open* (23).

### 2.2 Search strategy and sources

A comprehensive systematic literature search was conducted to identify all relevant published and unpublished studies. The electronic databases searched included PubMed, Embase, Web of Science, the Cochrane Library, China National Knowledge Infrastructure (CNKI), China Biomedical Literature Database (CBM), and Wanfang Data. All databases were searched from their inception to March 31, 2026. The search strategy combined controlled vocabulary (e.g., MeSH, Emtree) with free-text keywords. Search terms were constructed around three core concepts: (1) cardiac arrest; (2) extracorporeal membrane oxygenation/extracorporeal cardiopulmonary resuscitation (ECMO/ECPR); and (3) therapeutic hypothermia/targeted temperature management. These terms were combined using Boolean operators (AND, OR), with the syntax adapted to the specific requirements of each database. The full, reproducible search strategy for each database is detailed in Supplementary Appendix 1. Furthermore, to ensure comprehensive literature retrieval, the following supplementary search methods were employed: (1) searching Google Scholar; (2) searching the grey literature database OpenGrey; (3) manually screening the reference lists of all included studies; and (4) forward citation tracking of included studies.

### 2.3 Inclusion and exclusion criteria

#### 2.3.1 Inclusion criteria

The inclusion criteria for this study were formulated based on the Population, Intervention, Comparator, Outcomes, and Study designs (PICOS) framework as follows:

(1) Population: Adult patients (≥ 18 years of age) who underwent extracorporeal cardiopulmonary resuscitation (ECPR) for either in-hospital or out-of-hospital cardiac arrest.

(2) Intervention: Application of therapeutic hypothermia, typically defined as a target temperature between 32°C and 36°C, during or following ECPR.

(3) Comparator: Patients who underwent ECPR but did not receive active cooling. The control group could consist of normothermia management (e.g., a target temperature ≥ 36.5°C) or measures solely for fever prevention or treatment.

(4) Outcomes: Studies reporting on at least one of the following outcomes, including survival rate, favorable neurological outcome, and major complications associated with ECPR or therapeutic hypothermia (e.g., bleeding, infection, arrhythmias).

(5) Study designs: RCTs, prospective or retrospective cohort studies, and case-control studies.

#### 2.3.2 Exclusion criteria

The exclusion criteria were as follows:

(1) Publication type: Reviews, commentaries, editorials, case reports, conference abstracts, and letters to the editor.

(2) Subjects: Animal experiments or non-human studies.

(3) Data availability: Duplicate publications, studies from which relevant data could not be extracted, or those that did not report the outcomes of interest.

(4) Language: Studies published in languages other than English or Chinese.

(5) Study quality: For non-randomized studies, those judged to be at a “Critical” or “No information” overall risk of bias according to the Risk of Bias in Non-randomized Studies of Interventions (ROBINS-I) tool were excluded (24).

### 2.4 Study selection

**FIGURE 1:**
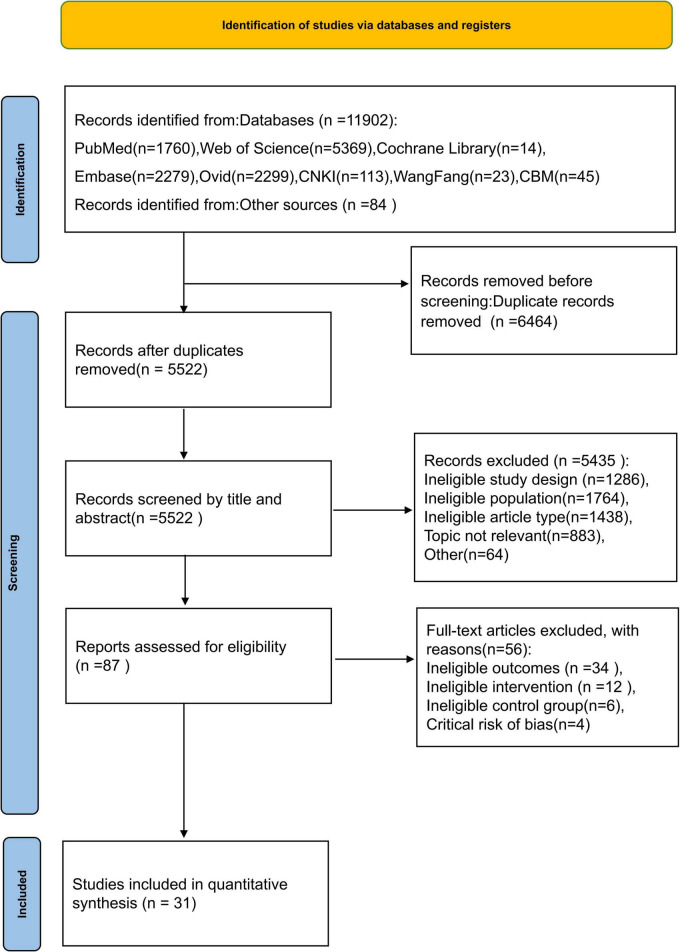
PRISMA flow diagram of the study selection process.

After completing all database searches, the retrieved records were imported into EndNote X9 (Clarivate Analytics, Philadelphia, PA, United States) for reference management. Duplicate records were first identified and removed using the software’s automated function, followed by a manual check. Subsequently, all deduplicated records were independently screened by two reviewers (B.M. and P.F.C.) in two stages. In the first stage, titles and abstracts were screened to exclude obviously irrelevant studies based on the predefined eligibility criteria. For studies deemed potentially eligible in this initial stage, their full texts were obtained for a detailed review in the second stage. The final decision on inclusion or exclusion for each article was then made by the two reviewers based on the complete inclusion and exclusion criteria. Throughout the screening process, any disagreements were resolved through discussion. At the full-text screening stage, the two reviewers (B.M. and P.F.C.) disagreed on 11 of 87 studies (12.6%); all such disagreements were resolved through discussion until consensus was reached, without the need for third-party adjudication. The results of the study selection process are illustrated in a PRISMA flow diagram (Figure 1).

### 2.5 Data extraction

Data were independently extracted by two reviewers (B.M. and P.F.C.) using a pre-piloted, standardized Excel form. Following extraction, the results were cross-checked, and any disagreements were resolved by discussion or, if necessary, through arbitration by a third reviewer (J.F.X.). The extracted data encompassed four main categories. Study characteristics included the first author, year of publication, country of origin, and study design. Baseline population characteristics comprised the sample size, age, sex, and details of the cardiac arrest. Intervention details covered the duration of ECPR and the specific temperature management protocols for both the intervention and control groups. Finally, outcome measures included survival rates at various time points, neurological outcomes assessed with the Cerebral Performance Category (CPC) or the modified Rankin Scale (mRS), and major ECPR-related complications. In cases where data were incomplete or missing, attempts were made to contact the corresponding authors for clarification.

### 2.6 Outcome measures

#### 2.6.1 Primary outcomes

The primary outcomes for this study were survival and favorable neurological function. Survival was assessed as a dichotomous variable (i.e., alive or deceased) at hospital discharge, 1 month (28/30 days), 3 months (90 days), and 6 months (180 days). Favorable neurological outcome was defined as the CPC score of 1 or 2, or the mRS score of 0 to 3 (25). The assessment time points for neurological outcome included hospital discharge, 1 month, and 3 months, as these were the periods for which data were consistently reported in the included studies.

#### 2.6.2 Secondary outcomes

Secondary outcomes comprised major complications relevant to the safety of therapeutic hypothermia. We prioritized complications recognized as being related to cooling—including hemorrhage, infection, and arrhythmia—while also extracting other major complications reported in the included studies, such as limb ischemia, acute kidney injury, and acute liver injury. The specific complications available for analysis were determined by the reporting of the included studies. As the definitions of these complications varied across studies, the definitions and diagnostic criteria reported by the authors of each original study were adopted and recorded directly.

### 2.7 Methodological quality assessment

The risk of bias for all included studies was independently assessed by two reviewers (B.M. and P.F.C.), with disagreements resolved by discussion or through arbitration by a third reviewer (J.F.X.). Initial agreement between the two reviewers was high; arbitration by the third reviewer was required for 4 of the 35 included studies (11.4%). The assessment tool was selected based on study design. For RCTs, the Cochrane Risk of Bias 2.0 (RoB 2.0) tool was used to evaluate five core domains: (1) bias arising from the randomization process, (2) bias due to deviations from intended interventions, (3) bias due to missing outcome data, (4) bias in measurement of the outcome, and (5) bias in selection of the reported result (26). Each domain was judged as “Low risk,” “Some concerns,” or “High risk,” leading to an overall risk of bias judgment for the study (26). For non-randomized studies, the ROBINS-I tool was employed to assess seven domains: (1) bias due to confounding, (2) bias in selection of participants into the study, (3) bias in classification of interventions, (4) bias due to deviations from intended interventions, (5) bias due to missing data, (6) bias in measurement of outcomes, and (7) bias in selection of the reported result (24). The risk of bias for each domain was rated as “Low,” “Moderate,” “Serious,” “Critical,” or “No information.” In accordance with the recommendations of the ROBINS-I development team, studies judged to be at a “Critical” risk of bias or for which there was “No information” were excluded from the analysis (24). The results of the risk of bias assessment were visualized using the robvis web app, providing a clear distribution of the risk of bias for each study across the assessed domains (27).

### 2.8 Data synthesis and statistical analysis

Data synthesis was performed using a random-effects model (DerSimonian–Laird method). This model was chosen given the anticipated clinical heterogeneity among the included studies in terms of design, population, and interventions. For dichotomous outcomes, such as survival rate and favorable neurological outcome, Risk Ratios (RR) were calculated and pooled as the primary effect measure, with pooling conducted on the log-transformed scale. To complement the relative effect estimates and to convey the clinical magnitude of the associations on an absolute scale, Risk Differences (RD) were also calculated and pooled for the primary outcomes (survival and favorable neurological outcome) using the Mantel–Haenszel method. For the secondary outcome (ECPR-related complications), only RR were reported. All pooled effect sizes were presented with their corresponding 95% confidence intervals (CI).

For observational studies, because covariate-adjusted estimates for the comparison of interest were not available across a sufficient number of studies, unadjusted (crude) event rates were used for quantitative synthesis. Accordingly, the pooled results from observational studies were interpreted as unadjusted associations rather than causal effects.

Statistical heterogeneity between studies was assessed using Cochran’s Q test and the *I*^2^ statistic. A significance level of *P* < 0.10 was set for the Q test. The *I*^2^ statistic was interpreted according to the following criteria: < 50% as low heterogeneity, 50–75% as moderate heterogeneity, and > 75% as high heterogeneity (28). For outcomes where the *I*^2^ value exceeded 75% or when the number of included studies was insufficient to support a meta-analysis, a narrative synthesis was conducted in accordance with the Synthesis Without Meta-analysis (SWiM) guidelines (28).

Potential sources of heterogeneity were explored through subgroup analyses based on the type of cardiac arrest and the TTM strategy. The robustness of the results was evaluated via two prespecified sensitivity analyses: a leave-one-out method and the application of a fixed-effect model. For outcomes comprising 10 or more studies, publication bias was investigated. This assessment involved visual inspection of contour-enhanced funnel plots for asymmetry, supplemented by quantitative evaluation with Egger’s regression and Begg’s rank correlation tests. *P* < 0.05 was taken to indicate significant publication bias. All meta-analyses were performed in Review Manager (RevMan, v5.3). The assessment of publication bias and all associated graphics were generated using R software (v4.4.3).

### 2.9 GRADE assessment

In this study, the certainty of evidence for each key outcome was systematically assessed using the Grading of Recommendations Assessment, Development and Evaluation (GRADE) framework (29). The core of this assessment involved adjusting an initial evidence rating based on an evaluation of prespecified downgrading and upgrading factors to determine the final certainty grade (29). The entire workflow was managed and documented using the GRADEpro GDT online platform.

## 3 Results

### 3.1 Literature search and selection results

The initial systematic literature search identified 11,986 records. After duplicate removal, 5,522 articles underwent title and abstract screening, leaving 87 for full-text assessment. Of these, 56 were excluded for either failing to meet eligibility criteria (*n* = 52) or being assessed at a “critical” risk of bias with the ROBINS-I tool (*n* = 4). This process resulted in 31 studies being included in the final analysis (30–60) (Figure 1).

### 3.2 Characteristics of included studies

A total of 31 studies (30–60) involving 6,184 patients who underwent ECPR, were included. Published between 2013 and 2026, the studies were predominantly observational, comprising 23 retrospective cohort studies (33–35, 37–43, 46–50, 52–57, 59, 60) and 5 prospective cohort studies (30–32, 45, 58), alongside 3 RCTs (36, 44, 51). Geographically, the evidence was concentrated in Asia, with studies from Japan (*n* = 10) (30, 32, 33, 40, 45, 46, 49, 53, 57, 59); South Korea (*n* = 9) (31, 34, 35, 39, 41–43, 47, 54); China (*n* = 4) (44, 55, 56, 60); and Singapore (*n* = 2) (36, 38), with the remainder from Europe (*n* = 3) (48, 51, 58), North America (*n* = 1) (50), and Australia (*n* = 1) (37), in addition to one international multi-center registry (52).

The patient population varied, with 16 studies (30–32, 34, 35, 40, 46, 47, 49, 53–55, 57–60) investigating OHCA cases exclusively, while 15 studies (33, 36–39, 41–45, 48, 50–52, 56) included both OHCA and IHCA patients. Across studies, the reported median or mean patient age ranged from 45.9 to 66.0 years, and the proportion of males ranged from 63.0 to 90.4%. Regarding outcomes, survival at discharge was reported in 16 studies (31–33, 36–39, 41–44, 48, 54, 56, 57, 60) and neurological outcome at discharge in 18 studies (32, 36–46, 49, 50, 54, 55, 57, 60); data on long-term outcomes were limited. ECPR-related complications were reported in 8 studies (36, 38, 41, 44, 48, 51, 52, 60). Detailed characteristics of all included studies are summarized in Table 1. Among the included studies, only 1 study (54) reported a covariate-adjusted estimate for therapeutic hypothermia versus control. Adjusted pooling was therefore not feasible, and crude event rates were used as prespecified.

**TABLE 1:** General characteristics of the included studies (*n* = 31).

Study	Region	Study design	Population	Age (years)	Male (%)	N (T/C)	Therapeutic hypothermia	Control strategy	Outcomes	Neuro. scale	Maximum follow-up period
Maekawa et al. (30)	Japan	Prosp. cohort	OHCA	54 (IQR: 47–60)	84.6	26/26	34°C for 48–96 h	Targeted normothermia	➂➆	CPC	3 months
Kim et al. (31)	Korea	Prosp. cohort	OHCA	53 (IQR: 41–68)	74.5	14/38	33°C for 24 h	Targeted normothermia	➀➆	CPC	3 months
Sakamoto et al. (32)	Japan	Prosp. cohort	OHCA	56.3^1^	90.4	231/29	32–34°C for ≥ 24 h	Targeted normothermia	➀➄	CPC	6 months
Kagawa et al. (33)	Japan	Retro. cohort	OHCA IHCA	62 (IQR: 52–72)	73.6	48/39	34–36°C for 24–48 h	Targeted normothermia	➀➆	CPC	3 months
Choi et al. (34)	Korea	Retro. cohort	OHCA	57.7 ± 6.2	70.0	6/4	33°C for 24 h	Targeted normothermia	➁➅	CPC	1 month
Lee et al. (35)	Korea	Retro. cohort	OHCA	55 (IQR: 40–68)	87.0	18/5	33–34°C for 24 h	Targeted normothermia	➁	CPC	1 month
Pang et al. (36)	Singapore	RCT	OHCA IHCA	45.9 ± 12.2 (T) 57.4 ± 7.0 (C)	81.0	9/12	34–36°C for 24 h	Targeted normothermia	➀➃➄➇	CPC	6 months
Dennis et al. (37)	Australia	Retro. cohort	OHCA IHCA	54 (IQR: 47–58)	73.0	15/22	33°C for 24 h	Targeted normothermia	➁➄	CPC	To discharge
Pang et al. (38)	Singapore	Retro. cohort	OHCA IHCA	49.9 ± 12.4	78.5	14/65	34°C for 24 h	Targeted normothermia	➀➄➇	CPC	13.4 months
Ryu et al. (39)	Korea	Retro. cohort	OHCA IHCA	59 (IQR: 46–70)	72.9	25/17	34–36°C for 48 h	Targeted normothermia	➀➄	CPC	To discharge
Yukawa et al. (40)	Japan	Retro. cohort	OHCA	59 (IQR: 48.5–64.5)	82.3	50/29	34–36°C for 24 h	Fever control	➄	CPC	To discharge
Kim et al. (41)	Korea	Retro. cohort	OHCA IHCA	55 ± 16.7	68.3	25/76	34–36°C for 24 h	Targeted normothermia	➀➄➇	CPC	To discharge
Han et al. (42)	Korea	Retro. cohort	OHCA IHCA	Survivors:40 ± 15 Nonsurvivors: 58 ± 14	74.0	26/74	33°C for 24 h	Targeted normothermia	➀➄	CPC	To discharge
Ryu et al. (43)	Korea	Retro. cohort	OHCA IHCA	62 (IQR: 51–73)	63.5	36/238	32–34°C for 24 h	Targeted normothermia	➀➄	CPC	To discharge
Pan et al. (44)	China	RCT	OHCA IHCA	52.1 ± 16.0	78.3	30/30	32–34°C for 24 h	Targeted normothermia	➀➄➇	CPC	To discharge
Kobata et al. (45)	Japan	Prosp. cohort	OHCA IHCA	57.5 (IQR: 43.25–66)	82.0	18/4	34–36°C for 24 h	Targeted normothermia	➄	CPC	6 months
Otani et al. (46)	Japan	Retro. cohort	OHCA	64 (IQR: 50–72)	82.0	33/123	32–34°C^2^	Targeted normothermia	➄	CPC	To discharge
Kim et al. (47)	Korea	Retro. cohort	OHCA	60.5 ± 12.2	88.0	38/146	32–34°C for 12–24 h	Fever control	➁	CPC	1 month
Mecklenburg et al. (48)	Germany	Retro. cohort	OHCA IHCA	T: 48 (IQR: 39–57) C: 55 (IQR: 45–62)	69.7	36/30	32–34°C for 26 h	Fever control	➀➁➇	NR	1 month
Yamada et al. (49)	Japan	Retro. cohort	OHCA	56 (IQR: 46–66)	86.0	202/66	34–36°C2	Fever control	➄	NR	1 month
Al-Kawaz et al. (50)	USA	Retro. cohort	OHCA IHCA	T: 61 (IQR: 50.5–73) C: 59.5 (IQR: 49–67.5)	63.0	10/29	34–36°C for 24 h	Targeted normothermia	➄	mRS	To discharge
Levy et al. (51)	France	RCT	OHCA IHCA	T: 57 ± 12 C: 59 ± 12	75.7	168/166	33–34°C for 24 h	Targeted normothermia	➁➃➇	CPC	6 months
Nakashima et al. (52)	International*	Retro. cohort	OHCA IHCA	56 (IQR: 44–65)	72.0	849/662	34–36°C for 12–48 h	Fever control	➂➇	NR	3 months
Sakurai et al. (53)	Japan	Retro. cohort	OHCA	T: 57 (IQR: 47–67) C: 62 (IQR: 49–71)	84.7	471/506	32–34°C for 24–48 h	Targeted normothermia	➅	CPC	1 month
Kim et al. (54)	Korea	Retro. cohort	OHCA	50 (IQR: 41–60)	84.8	69/69	34–36°C for 12–24 h	Fever control	➀➄	CPC	To discharge
Shih et al. (55)	China	Retro. cohort	OHCA	56 (IQR: 45–64)	83.5	55/30	34–36°C2	Fever control	➄	CPC	To discharge
Yuan et al. (56)	China	Retro. cohort	OHCA IHCA	53.38 ± 15.05	75.8	385/263	34–36°C2	Fever control	➀	NR	To discharge
Ijuin et al. (57)	Japan	Retro. cohort	OHCA	63 (IQR: 52–70)	74.8	293/180	34–36°C2	Fever control	➀➄	CPC	To discharge
Pöss et al. (58)	Germany	Prosp. cohort	OHCA	66 (IQR: 57–75)	73.7	245/306	33–36°C for 12–24 h	Targeted normothermia	➅	CPC	16 months
Taira et al. (59)	Japan	Retro. cohort	OHCA	60 (IQR: 49–70)	76.8	36/227	32–36°C2	Fever control	➅	CPC	1 month
Lee et al. (60)	China	Retro. cohort	OHCA	59 (IQR: 49–66)	75.0	79/113	33°C for 24 h	Normothermia	➀➄➇	CPC	To discharge

RCT, Randomized controlled trial; Prosp. cohort, Prospective cohort; Retro. cohort, Retrospective cohort; T, Therapeutic hypothermia group; C, Control group; OHCA, Out-of-hospital Cardiac Arrest; IHCA, In-hospital Cardiac Arrest; IQR, interquartile range; CPC, Cerebral Performance Category; mRS, modified Rankin Scale; NR, Not reported; *Data for the study were obtained from the extracorporeal life support organization registry;

^1^Represents the mean age as provided in the original publication;

^2^Maintenance duration for targeted temperature management was not reported. Targeted Normothermia was defined as any strategy involving the active maintenance of core body temperature within a normal range; Fever control (also referred to as standard care or reactive management) was defined as the treatment of fever on an as-needed basis without a proactive temperature control protocol; ➀ Survival to hospital discharge; ➁ Survival at 1 month; ➂ Survival at 3 months; ➃ Survival at 6 months; ➄ Neurological outcome at hospital discharge; ➅ Neurological outcome at 1 month; ➆ Neurological outcome at 3 months; and ➇ Complications.

### 3.3 Quality assessment

**FIGURE 2:**
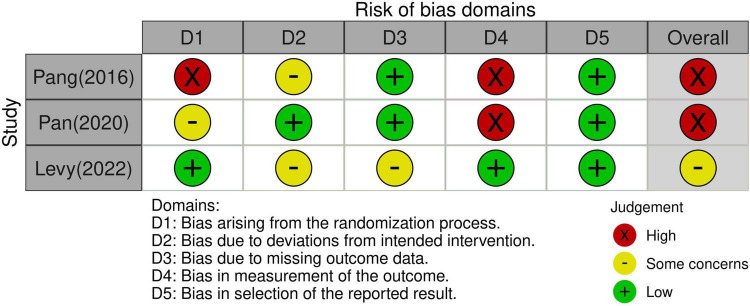
Risk of bias for randomized controlled trials (RoB 2.0).

**FIGURE 3:**
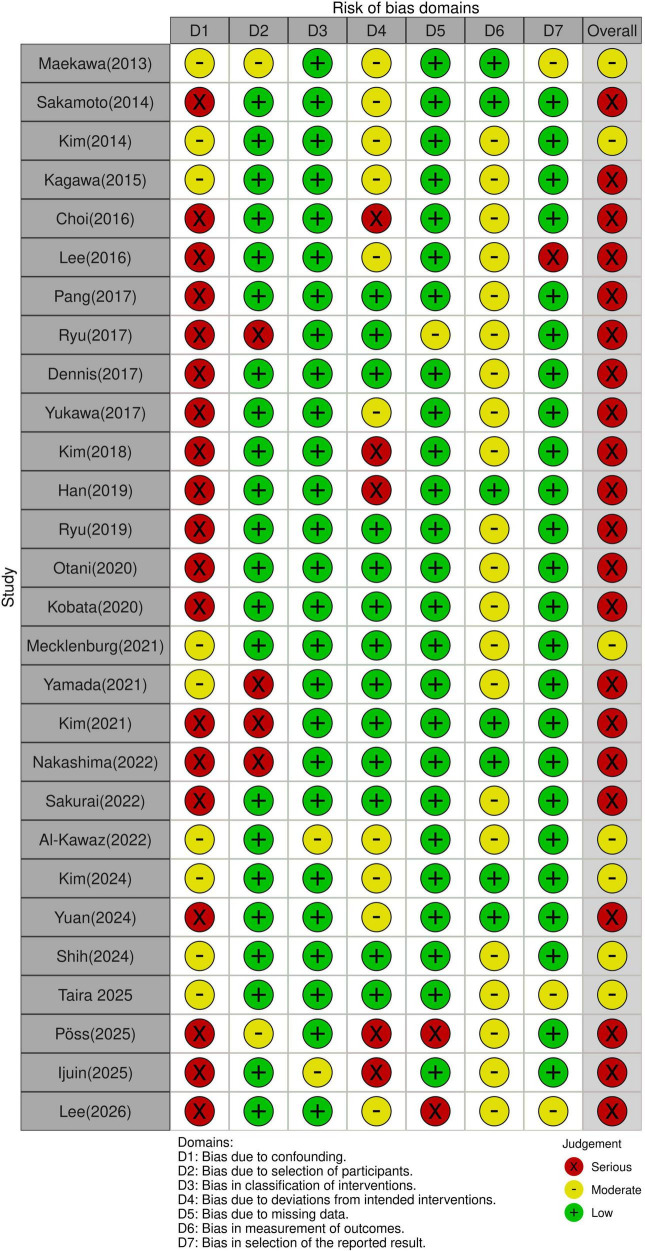
Risk of bias for non-randomized studies of interventions (ROBINS-I).

Of the 3 RCTs (36, 44, 51) assessed with the RoB 2.0 tool, 2 (36, 44) were rated at “high risk” of bias due to deficiencies in the randomization process and blinding. The other RCT (51) was rated as having “some concerns” due to issues with deviations from intended interventions and missing data arising from its open-label design. The remaining 28 non-randomized studies (30–35, 37–43, 45–50, 52–60) were assessed with the ROBINS-I tool and showed considerable overall risk of bias: 21 were “serious” (32–35, 37–43, 45–47, 49, 52, 53, 56–58, 60) and 7 “moderate” (30, 31, 48, 50, 54, 55, 59). Detailed risk-of-bias assessments for each study are presented in Figure 2 (RoB 2.0) and Figure 3 (ROBINS-I). The overall proportions of risk-of-bias judgments across domains are shown in Supplementary Figures 1, 2.

### 3.4 Meta-analysis results

#### 3.4.1 Primary outcomes

**FIGURE 4:**
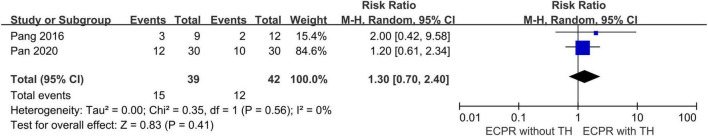
Forest plot of survival to discharge (RCTs).

**FIGURE 5:**
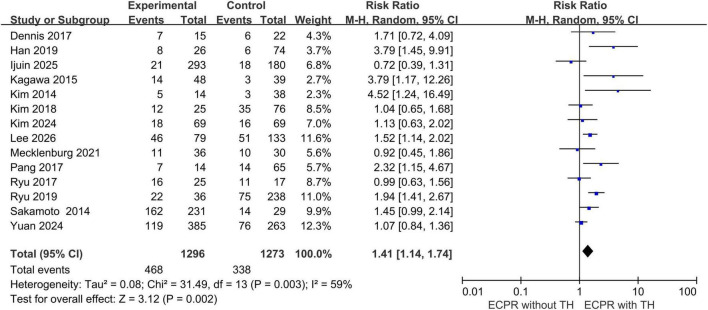
Forest plot of survival to discharge (Non-RCTs).

The meta-analysis for survival to hospital discharge included 16 studies (31–33, 36–39, 41–44, 48, 54, 56, 57, 60) (*n* = 2,650; 1,335 therapeutic hypothermia vs. 1,315 control). Because randomized and observational designs differ fundamentally in their susceptibility to confounding and selection bias, the two study types were analyzed separately rather than pooled into a single overall estimate, and randomized evidence was regarded as the primary basis for inference. In the RCTs (36, 44) (2 studies; *n* = 81; 39 therapeutic hypothermia vs. 42 control), therapeutic hypothermia was not associated with a statistically significant difference in survival (RR = 1.30, 95% CI: 0.70–2.40; *P* = 0.41; *I*^2^ = 0%) (Figure 4), and the absolute difference was likewise non-significant (RD = 0.10, 95% CI: −0.11 to 0.30; *P* = 0.35; *I*^2^ = 0%) (Supplementary Figure 3). In contrast, the observational studies (31–33, 37–39, 41–43, 48, 54, 56, 57, 60) (14 studies; *n* = 2,569; 1,296 therapeutic hypothermia vs. 1,273 control) showed a statistically significant association between therapeutic hypothermia and better survival (RR = 1.41, 95% CI: 1.14–1.74; *P* = 0.002), with substantial heterogeneity across studies (*I*^2^ = 59%; *P* = 0.003). In absolute terms, this corresponded to an RD of 0.12 (95% CI: 0.05–0.20; *P* = 0.001) (Figure 5 and Supplementary Figure 3). However, given the observational design, these estimates reflect association rather than a causal treatment effect and may be inflated by residual confounding and selection bias—for example, patients who survived long enough or had more favorable initial status may have been more likely to receive temperature management.

**FIGURE 6:**
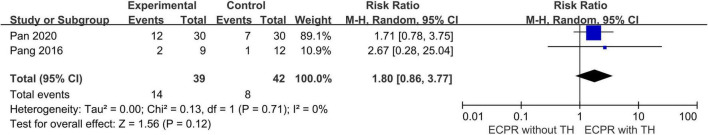
Forest plot of favorable neurological outcome at discharge (RCTs).

**FIGURE 7:**
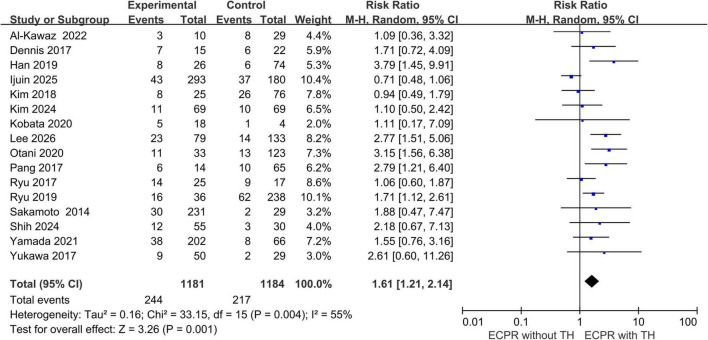
Forest plot of favorable neurological outcome at discharge (Non-RCTs).

The meta-analysis for favorable neurological outcome at discharge included 18 studies (*n* = 2,446; 1,220 therapeutic hypothermia vs. 1,226 control) (32, 36–46, 49, 50, 54, 55, 57, 60). Consistent with the survival to hospital discharge, RCTs and observational studies were analyzed separately rather than combined into a single overall estimate, and the randomized evidence was prioritized as the primary basis for inference. In the RCTs (36, 44) (2 studies; *n* = 81; 39 therapeutic hypothermia vs. 42 control), therapeutic hypothermia was not associated with a statistically significant difference in favorable neurological outcome (RR = 1.80, 95% CI: 0.86–3.77; *P* = 0.12; *I*^2^ = 0%), and the absolute difference likewise did not reach significance (RD = 0.16, 95% CI: −0.03 to 0.34; *P* = 0.10; *I*^2^ = 0%) (Figure 6 and Supplementary Figure 4). In contrast, the observational studies (32, 37–43, 45, 46, 49, 50, 54, 55, 57, 60) (16 studies; *n* = 2,365; 1,181 therapeutic hypothermia vs. 1,184 control) demonstrated a statistically significant association between therapeutic hypothermia and more favorable neurological outcomes (RR = 1.61, 95% CI: 1.21–2.14; *P* = 0.001), with moderate heterogeneity (*I*^2^ = 55%; *P* = 0.004). In absolute terms, this corresponded to an RD of 0.09 (95% CI: 0.04–0.15; *P* = 0.0009; *I*^2^ = 49%) (Figure 7 and Supplementary Figure 4).

Taken together, these design-stratified analyses indicate that the more favorable survival and neurological outcomes at discharge were derived primarily from observational data. However, the apparently favorable association observed in the observational studies was not confirmed by the available randomized evidence. This discordance between observational and randomized data should be interpreted cautiously and underscores the need for adequately powered RCTs before any firm conclusion regarding the efficacy of therapeutic hypothermia in ECPR can be drawn.

Stratified meta-analyses by follow-up duration are summarized in Table 2, with the corresponding forest plots provided in Supplementary Figures 5–14. Because the available studies at each individual time point originated almost exclusively from a single design type, the results are reported by study design rather than as combined estimates. In terms of survival outcomes, at 1 month, the evidence derived entirely from observational studies (34, 35, 47, 48) (4 studies; 98 therapeutic hypothermia vs. 185 control) and showed no significant association between therapeutic hypothermia and survival on either the relative or the absolute scale (RR = 0.91, 95% CI: 0.46–1.82, *P* = 0.79, *I*^2^ = 64%; RD = 0.00, 95% CI: −0.27 to 0.28, *P* = 0.98, *I*^2^ = 73%), with substantial between-study heterogeneity. In addition, 1 RCT (51) reported 1-month survival comparing moderate hypothermia (33–34 °C) with strict normothermia (36–37 °C); survival was higher in the moderate hypothermia group (58%) than in the normothermia group (49%), but the difference was not statistically significant. Similarly, the 3-month observational estimate (48, 52) (2 studies; 864 vs. 679) showed no difference (RR = 0.95, 95% CI: 0.73–1.24, *P* = 0.73, *I*^2^ = 14%; RD = −0.01, 95% CI: −0.10 to 0.08, *P* = 0.79, *I*^2^ = 18%). At 6 months, the 2 RCTs (36, 51) available (177 vs. 178) likewise demonstrated no significant difference in survival (RR = 1.37, 95% CI: 0.56–3.35, *P* = 0.49, *I*^2^ = 29%; RD = 0.08, 95% CI: −0.05 to 0.21, *P* = 0.23, *I*^2^ = 12%), with confidence intervals crossing the null on both scales. Thus, across all available follow-up time points, no significant association with survival was demonstrated. In terms of neurologic outcome, data were available only from observational studies, with no randomized evidence at these time points. At 1 month (34, 53, 58, 59) (4 studies; 540 vs. 771), therapeutic hypothermia was associated with a favorable neurological outcome on the relative scale (RR = 1.71, 95% CI: 1.12–2.64, *P* = 0.01, *I*^2^ = 9%), although the corresponding absolute difference was modest and only marginally significant, with the lower bound approaching the null (RD = 0.06, 95% CI: 0.00–0.12, *P* = 0.04, *I*^2^ = 25%). At 3 months (30, 31, 33) (3 studies; 88 vs. 103), both the relative and absolute estimates were larger (RR = 4.33, 95% CI: 1.87–9.99, *P* = 0.0006, *I*^2^ = 0%; RD = 0.23, 95% CI: 0.13–0.34, *P* < 0.0001, *I*^2^ = 0%).

**TABLE 2:** Meta-analysis of outcomes at different time points for ECPR patients with therapeutic hypothermia.

Outcome	Time point	Type of study	No. of patients (T/C)	RR (95% CI)	*P*-value	*I*^2^(RR)	RD (95% CI)	*P*-value	*I*^2^(RD)
Survival	1 month	Observational studies, *n* = 4 (34, 35, 47, 48)	98/185	0.91 (0.46–1.82)	0.79	64%	0.00 (−0.27 to 0.28)	0.98	73%
	3 months	Observational studies, *n* = 2 (48, 52)	864/679	0.95 (0.73–1.24)	0.73	14%	−0.01 (−0.10 to 0.08)	0.79	18%
	6 months	RCTs, *n* = 2 (36, 51)	177/178	1.37 (0.56–3.35)	0.49	29%	0.08 (−0.05 to 0.21)	0.23	12%
Neurological outcome	1 month	Observational studies, *n* = 4 (34, 53, 58, 59)	540/771	1.71 (1.12–2.64)	0.01	9%	0.06 (0.00–0.12)	0.04	25%
	3 months	Observational studies, *n* = 3 (30, 31, 33)	88/103	4.33 (1.87–9.99)	0.0006	0%	0.23 (0.13–0.34)	< 0.0001	0%

CI, confidence interval; ECPR, extracorporeal cardiopulmonary resuscitation; RR, risk ratio; RD, risk difference; T, Therapeutic hypothermia group; C,Control group. Pooled effect sizes were calculated using a random-effects model.

#### 3.4.2 Secondary outcomes

Meta-analyses of five major ECPR-related complications—hemorrhage, infection, limb ischemia, acute kidney injury, and liver injury—showed no significant association with therapeutic hypothermia in either randomized or observational studies (all *P* > 0.05). Heterogeneity was low among the RCTs (*I*^2^ = 0–17%) and low to moderate among the observational studies (*I*^2^ = 0–50%). Detailed data are provided in Table 3, and the corresponding forest plots are shown in Supplementary Figures 15–23.

**TABLE 3:** Meta-analysis of the effect of therapeutic hypothermia on ECPR-related complications in ECPR patients.

Complication	Type of Study	No. of Patients (T/ C)	RR (95% CI)	*P*-value	*I* ^2^
Hemorrhage	RCTs, *n* = 2 (36, 51)	177/178	0.97(0.76–1.25)	0.82	0%
	Observational studies, *n* = 5 (38, 41, 48, 52, 60)	1003/966	0.99(0.74–1.32)	0.94	49%
Infection	RCTs, *n* = 3 (36, 44, 51)	207/208	0.94(0.68–1.31)	0.72	17%
	Observational studies, *n* = 3 (48, 52, 60)	964/825	0.92(0.54–1.55)	0.75	50%
Limb ischemia	RCTs, *n* = 2 (36, 44)	39/42	1.09(0.52–2.27)	0.82	0%
	Observational studies, *n* = 3 (38, 41, 52)	888/803	1.01(0.71–1.42)	0.96	0%
Acute kidney injury	RCTs, *n* = 2 (36, 44)	39/42	0.99(0.77–1.28)	0.95	0%
	Observational studies, *n* = 2 (38, 48)	50/95	1.21(0.78–1.87)	0.40	40%
Liver injury	Observational studies, *n* = 2 (38, 48)	50/95	0.83(0.67–1.03)	0.09	0%

CI, confidence interval; ECPR, extracorporeal cardiopulmonary resuscitation; RR, risk ratio; T, Therapeutic hypothermia group; C, Control group. Pooled effect sizes were calculated using a random-effects model.

Several additional complications of particular relevance to therapeutic hypothermia were reported in single studies only and could not be pooled. Mecklenburg et al. (48) reported a higher incidence of lung injury with therapeutic hypothermia (83.3% vs. 30.0%; *P* < 0.001) but no significant difference in multiple organ failure (88.9% vs. 70.0%; *P* = 0.06). Nakashima et al. (52) reported a higher incidence of seizures (5.2% vs. 2.1%; *P* = 0.002) but no difference in hemodynamic instability (27.7% vs. 27.9%; *P* = 0.94). In addition, arrhythmia was reported in 1 RCT (36) and 1 retrospective cohort study (52); neither showed a significant between-group difference. Pang et al. (36) reported rates of 22.2% with therapeutic hypothermia and 16.7% with normothermia (*P* = 0.748), and Nakashima et al. (52) reported rates of 22 and 21%, respectively (*P* = 0.752).

### 3.5 Subgroup analysis

Given the limited number of eligible RCTs, and because pooling observational studies together with RCTs would render the combined estimates susceptible to confounding and selection bias, the subgroup analyses in this study were restricted to observational studies. Firstly, a subgroup analysis was conducted according to patient population (mixed OHCA/IHCA vs. pure OHCA). For survival to hospital discharge, this comparison included 14 studies (31, 33, 37–39, 41–43, 48, 54, 56, 57, 60) (mixed OHCA/IHCA: 9 studies, 610 patients with therapeutic hypothermia vs. 824 without; pure OHCA: 5 studies, 686 vs. 449), and the test for subgroup interaction was not significant (P for interaction = 0.63). For favorable neurological outcome, the comparison included 16 studies (32, 37–43, 45, 46, 49, 50, 54, 55, 57, 60) (mixed OHCA/IHCA: 8 studies, 169 vs. 525; pure OHCA: 8 studies, 1,012 vs. 659), again with no significant interaction (*P* for interaction = 0.75). The non-significant interaction tests provide no evidence that patient population modified the association; however, given the limited number of studies and the low statistical power of interaction tests in meta-analysis, effect modification by patient population cannot be excluded (Supplementary Figures 24, 25).

**FIGURE 8:**
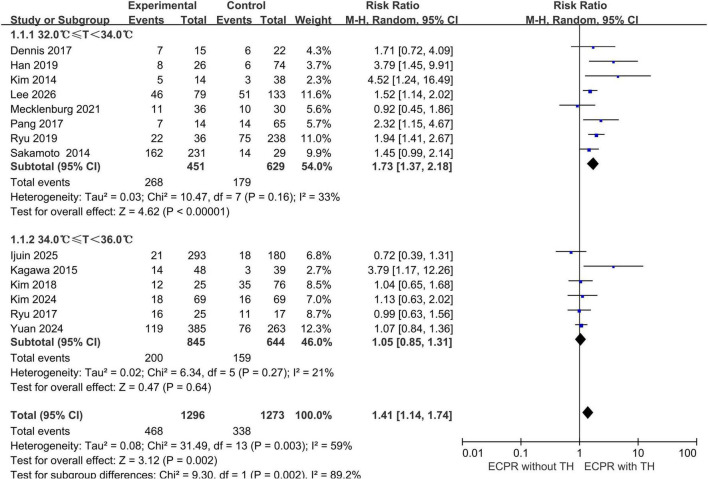
Forest plot of subgroup analysis of survival rates at discharge under different targeted temperature management strategies.

**FIGURE 9:**
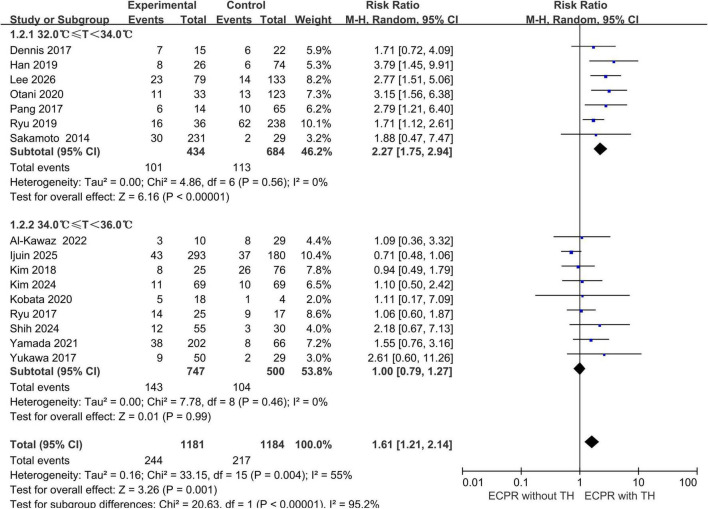
Forest plot of subgroup analysis of neurological function outcomes at discharge under different targeted temperature management strategies.

Secondly, the included studies were stratified by target temperature into a “moderate therapeutic hypothermia” group (32.0 °C ≤ T < 34.0 °C) and a “mild therapeutic hypothermia” group (34.0 °C ≤ T < 36.0 °C). For survival to hospital discharge, the moderate group comprised 8 studies (31, 32, 37, 38, 42, 43, 48, 60) (451 vs. 629) and the mild group 6 studies (33, 39, 41, 54, 56, 57) (845 vs. 644). Moderate therapeutic hypothermia was associated with significantly better survival (RR = 1.73, 95% CI: 1.37–2.18, *P* < 0.001, *I*^2^ = 33%), whereas no significant association was observed in the mild hypothermia group (RR = 1.05, 95% CI: 0.85–1.31, *P* = 0.64, *I*^2^ = 21%) (Figure 8). For favorable neurological outcome, the moderate group comprised 7 studies (32, 37, 38, 42, 43, 46, 60) (434 vs. 684) and the mild group 9 studies (39–41, 45, 49, 50, 54, 55, 57) (747 vs. 500). In the moderate therapeutic hypothermia group, therapeutic hypothermia was associated with a more favorable neurological outcome at discharge (RR = 2.27, 95% CI = 1.75–2.94, *P* < 0.00001, *I*^2^ = 0%), whereas no significant association was observed in the mild therapeutic hypothermia group (RR = 1.00, 95% CI = 0.79–1.27, *P* = 0.46, *I*^2^ = 0%) (Figure 9). The significant test for subgroup interaction (*P* for interaction = 0.002 and < 0.00001, respectively), with most of the overall heterogeneity attributable to differences between temperature subgroups (between-subgroup *I*^2^ = 89.2 and 95.2%, respectively), suggesting that target temperature is an important source of heterogeneity and that this association may be largely confined to the moderate temperature range.

### 3.6 Sensitivity analysis

Two sensitivity analysis methods were implemented to evaluate the robustness of the meta-analysis results. First, all outcome measures were re-analyzed by switching between the random-effects and fixed-effect models. The results indicated that the pooled effect sizes and their statistical significance were highly consistent between the two models, suggesting that the main conclusions are robust to the choice of analytical model (Supplementary Figures 26–37). Second, the leave-one-out sensitivity analysis showed that the sequential removal of each study did not substantially alter the pooled effect estimates or their significance for any primary outcome, and the direction of the effect remained stable. Furthermore, this analysis also identified individual studies as the primary sources of heterogeneity for specific outcomes. Specifically, the removal of one study (57) reduced heterogeneity for favorable neurological outcome at discharge from *I*^2^ = 55–22%. Similarly, heterogeneity for survival at 1 month was reduced from *I*^2^ = 64–34% after another study (60) was removed. The study by Mecklenburg et al. (48) was also identified as the main contributor to heterogeneity for hemorrhage and infection events, respectively (Supplementary Figures 38–41). Critically, even after the aforementioned studies contributing to high heterogeneity were removed, the direction and statistical significance of the pooled effects for all primary outcomes remained unchanged. To further examine the influence of the most heterogeneity-inducing studies, we additionally removed the two largest contributors to heterogeneity for each pooled outcome simultaneously. For favorable neurological outcome at discharge, the simultaneous exclusion of Ijuin et al. (57). and Kim et al. (41). reduced heterogeneity from *I*^2^ = 55% to *I*^2^ = 9%, while the pooled effect remained significant and directionally unchanged (RR = 1.84, 95% CI = 1.48–2.30). For survival at 1 month, the simultaneous exclusion of Lee et al. (35). and Mecklenburg et al. (48). reduced heterogeneity from *I*^2^ = 64% to *I*^2^ = 0% (RR = 1.57, 95% CI = 0.85–2.92). This indicates that, while the association with favorable neurological outcome was robust to the exclusion of the most influential studies, the association with survival at 1 month was more sensitive to individual studies and should therefore be interpreted with caution (Supplementary Figures 42–43).

### 3.7 Publication bias

Publication bias was assessed for outcomes that included more than 10 studies. The assessment involved visual inspection of contour-enhanced funnel plots, supplemented by quantitative evaluation using Begg’s rank correlation test and Egger’s linear regression test. For survival to hospital discharge, the funnel plot exhibited visual asymmetry, suggesting potential publication bias (Supplementary Figure 44). However, the results of both Begg’s test (*Z* = 1.59, *P* = 0.11) and Egger’s test (*t* = 1.27, *P* = 0.23) were not statistically significant. Nevertheless, considering the potential for low statistical power of these tests with a limited number of studies, the possibility of publication bias for this outcome could not be fully ruled out based on the visual inspection. For favorable neurological outcome at discharge, which was reported by more than 10 studies, neither Begg’s rank correlation test (*Z* = 0.63, *P* = 0.53) nor Egger’s linear regression test (*t* = 1.59, *P* = 0.13) revealed significant funnel-plot asymmetry. The contour-enhanced funnel plot showed that most studies fell within the non-significance contours, indicating a low likelihood of substantial publication bias. Nevertheless, given the moderate heterogeneity (*I*^2^ = 55%), the slight asymmetry may partly reflect between-study heterogeneity rather than publication bias alone, and the findings should be interpreted with caution (Supplementary Figure 45).

### 3.8 Certainty of evidence

The certainty of evidence was assessed using GRADE. Four RCT safety outcomes (hemorrhage, infection, limb ischemia, and acute kidney injury) were rated as low certainty; all remaining outcomes were very low certainty (detailed in Supplementary Table 1).

## 4 Discussion

In this systematic review and meta-analysis, we set out to determine whether the addition of therapeutic hypothermia to ECPR is associated with better survival and more favorable neurological outcomes. Because randomized and observational designs differ fundamentally in their vulnerability to confounding and selection bias, all analyses were stratified by study design, with the randomized evidence regarded as the primary basis for inference. On this basis, our principal finding is that the available randomized evidence does not demonstrate a statistically significant benefit of therapeutic hypothermia for either survival to discharge or favorable neurological outcome at discharge. This absence of benefit extended to later time points: no significant effect on survival was seen at 1, 3, or 6 months, and although observational data suggested more favorable neurological outcomes at 1 and 3 months, these estimates rested on small, unadjusted samples with wide confidence intervals. These observational associations were based on crude, unadjusted event rates and likely reflect residual confounding and selection bias rather than a causal effect. With respect to safety, therapeutic hypothermia was not associated with any of the five major ECPR-related complications—hemorrhage, infection, limb ischemia, acute kidney injury, or liver injury—in either randomized or observational studies. Taken together, all of the apparently beneficial associations were confined to observational data and were not corroborated by randomized evidence; they should therefore be interpreted with caution.

These findings can be placed in the context of previous reviews, although the comparison must be interpreted in light of the type of evidence on which each conclusion rests. In the 2020 systematic review by Chen et al. (20), therapeutic hypothermia was reported to be associated with higher survival and more favorable neurological outcomes in ECPR patients. It should be noted, however, that this conclusion—as with the favorable associations observed in the present observational stratum—was driven largely by unadjusted observational data and therefore reflects an association rather than evidence of a treatment effect. When interpretation is restricted to the pooled randomized evidence, as it should be, no such benefit is established by either the present analysis or the randomized data available to date. A further difference concerns the survival endpoint: “30-day” and “at-discharge” survival were pooled into a single subgroup by Chen et al. (20), whereas in the present analysis survival at discharge, 1 month, and 3 months were treated as separate endpoints. This methodological distinction may explain why a survival association reported in the earlier review was not reproduced; because these are indirect between-study comparisons of largely unadjusted data, however, they should be read as differences in reported associations rather than as differences in treatment effect. On the other hand, our findings also relate to the 2023 review by Bian et al. (61), in which short-term associations were described and a target range of 33–35 °C was proposed. That recommendation was based on a descriptive summary of the included studies rather than a formal statistical evaluation of target temperature (61). In the present subgroup analysis, the apparent association was most evident in the 32.0–34.0 °C range and was absent at 34–36 °C, a difference that may partly explain why no clear between-subgroup contrast emerged in the earlier pooled estimate. This comparison should nonetheless be regarded as hypothesis-generating only, as it is derived entirely from unadjusted observational subgroups and does not constitute evidence that a particular target temperature confers a differential treatment benefit. Taken together, the present analysis refines rather than overturns these earlier reviews, while underscoring that the apparent associations reported across this literature have not been confirmed by randomized evidence.

In previous studies, a benefit of therapeutic hypothermia has been demonstrated in selected populations. In the HYPERION trial (18), a target of 33 °C was associated with a more favorable neurological outcome compared with 37 °C in a more severely ill, predominantly non-shockable cohort. This finding is biologically plausible, as the metabolic suppression, attenuation of excitotoxicity, and anti-inflammatory effects associated with lower target temperatures may be most relevant in patients sustaining severe ischemia–reperfusion injury (21, 62). Such a benefit, however, has not been observed more broadly. The TTM1 (63) and TTM2 (14) trials, conducted in heterogeneous OHCA populations of generally lower injury severity, were both neutral, suggesting that therapeutic hypothermia confers little additional protection once adequate temperature control is achieved. Consistent with these latter trials, the pooled randomized estimate in the present analysis did not demonstrate a benefit of therapeutic hypothermia in ECPR patients. This randomized evidence, nonetheless, was limited to a few trials with small samples. Although a favorable association was observed in the pooled observational data, such data were unadjusted and the overall certainty of evidence was very low, precluding any conclusion regarding a treatment effect. Adequately powered randomized trials in ECPR populations are therefore required before therapeutic hypothermia can be recommended.

The long-term survival and neurological status of cardiac arrest survivors are of equal clinical importance. To explore how the apparent influence of therapeutic hypothermia might evolve over time, survival and favorable neurological outcome were therefore examined as separate endpoints at successive time points. For survival, no association with therapeutic hypothermia was evident at any of the three time points assessed (1, 3, and 6 months). For favorable neurological outcome, by contrast, a positive association was observed at both 1 and 3 months. This divergence between the long-term survival and neurological outcomes may relate to the mechanisms through which hypothermia is thought to act. Its putative neuroprotective effects—including reduction of the cerebral metabolic rate (64), attenuation of glutamate-mediated excitotoxicity (15), and stabilization of the blood–brain barrier (65)—would be expected to influence the quality of recovery among survivors rather than the probability of survival itself. Survival, meanwhile, is shaped to a greater degree by competing determinants such as underlying cardiovascular disease and non-cardiac organ failure (66, 67), which lie largely beyond the reach of temperature management. A neuroprotective effect might therefore manifest as more favorable functional status without translating into a measurable long-term survival gain. This interpretation, however, must be qualified, as the neurological associations were derived exclusively from unadjusted observational studies of very low certainty. Whether therapeutic hypothermia genuinely leads to more favorable neurological recovery in ECPR survivors, and whether such an effect accumulates over time, can only be resolved by adequately powered randomized trials reporting prospectively defined functional outcomes at multiple time points.

The invasive nature of ECPR carries an inherent risk of ECPR-related complications, most commonly hemorrhage, infection, limb ischemia, acute kidney injury, and arrhythmias (65). This raises a clinical consideration when therapeutic hypothermia is contemplated, as the intervention itself has been associated with a broadly similar spectrum of adverse effects (65). A relevant safety question is therefore whether adding hypothermia to ECPR is associated with an increase in these risks. Our meta-analysis did not detect a statistically significant difference in the incidence of major complications between patients who did and did not receive therapeutic hypothermia. This finding, however, should be interpreted with considerable caution, and the absence of a statistically significant difference should not be taken as evidence of safety. Because the analysis was based predominantly on observational studies and may have been underpowered owing to sample-size limitations, it cannot reliably exclude a modest but clinically important difference in harm.

These findings carry several implications for future research. Although the favorable association observed in the observational studies was not confirmed by the pooled randomized data, this degree of uncertainty is itself broadly consistent with the joint EuSEM and ESAIC statement (21), and suggests that the role of therapeutic hypothermia in the ECPR subgroup merits continued and more rigorous investigation rather than outright dismissal. Beyond this, the exploratory subgroup analysis may help to refine the direction of such investigation. The apparent association observed within the 32–34 °C range, with no corresponding signal at 34–36 °C, should be regarded only as a hypothesis worthy of prospective testing. By extension, it suggests that both future trials and clinical practice should move beyond a simple binary choice towards individualized patient stratification, ideally guided by multimodal assessment of brain injury (68). An adequately powered, multicenter randomized trial with prospective stratification by prognostic factors and standardized outcome ascertainment is therefore warranted.

The findings of this study should be interpreted within the framework of several important limitations. First, the evidence base is predominantly composed of observational studies. This design carries an inherent risk of bias that cannot be fully mitigated, thereby reducing the certainty of the final effect estimates. Importantly, the observational studies were pooled using unadjusted effect estimates; consequently, the observational subgroup results are susceptible to bias from unmeasured and residual confounding as well as from selection bias, and should be interpreted with this caveat in mind. This concern underscores why we analyzed and reported the randomized and observational evidence separately, and why our conclusions are framed primarily around the design-stratified findings rather than the combined estimates. Second, substantial heterogeneity was observed across the included studies, mandating a cautious interpretation of any pooled results. This statistical heterogeneity is likely driven by underlying clinical diversity in temperature targets, cooling methods, and rewarming protocols, which precludes the formulation of a standardized, one-size-fits-all therapeutic regimen. Finally, the search strategy was restricted to publications in English and Chinese, potentially introducing language bias. The possibility that relevant studies with contradictory findings published in other languages were omitted cannot be fully excluded.

## 5 Conclusion

In conclusion, in patients undergoing ECPR, the available randomized evidence does not demonstrate better survival or more favorable neurological outcomes with therapeutic hypothermia, whereas the apparently favorable associations seen in observational studies were based on crude, unadjusted data and are at high risk of confounding and selection bias. Furthermore, therapeutic hypothermia was not associated with an increased risk of major complications. Overall, the certainty of evidence is very low, and no firm conclusion regarding the efficacy of therapeutic hypothermia in ECPR can yet be drawn. Adequately powered randomized controlled trials are needed to determine whether therapeutic hypothermia—and in particular a moderate target temperature—confers a genuine causal benefit and to define its role in clinical practice.

## Data Availability

The original contributions presented in this study are included in this article/Supplementary material, further inquiries can be directed to the corresponding author.
