# Supplementary material for: Survival and neurological outcome in patients treated with extracorporeal membrane oxygenation and therapeutic hypothermia: an updated systematic review and meta-analysis
Source: Front Med (Lausanne). 2026 Jul 10;13:1882223. doi: 10.3389/fmed.2026.1882223 (PMC13395984; doi:10.3389/fmed.2026.1882223)
Supplement: Supplementary file 1 [file Data_Sheet_1.pdf]

Survival and Neurological Outcome in Patients Treated with Extracorporeal Membrane Oxygenation and Therapeutic Hypothermia: An Updated Systematic Review and Meta-Analysis Supplemental Material

Table of Contents

Risk of Bias Assessment 3
Forest plots of the meta-analysis at different time points of the primary outcome 5
Forest plots of the meta-analysis of different complications in the secondary outcomes 17
Subgroup analysis 26
Sensitivity analysis: fixed effects model 28
Sensitivity analysis: leave-one-out method 40
Publication Bias 46
Certainty of Evidence 48
Appendix A: Search strategy 51
Appendix B: PRISMA Checklist 55

Risk of Bias Assessment

Supplemental Figure 1 Risk of bias summary for randomized controlled trials (RoB 2.0).

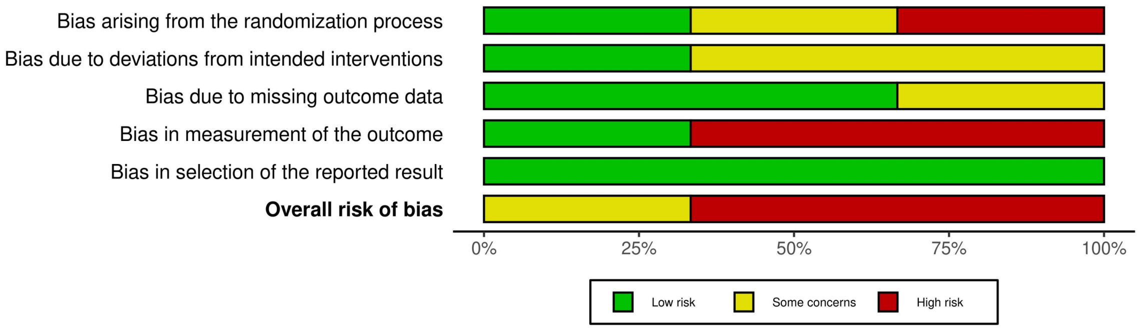

The graph shows the proportion of studies rated as low risk, some concerns, and high risk for each bias domain according to the Cochrane RoB 2.0 tool.

Supplemental Figure 2 Risk of bias summary for non-randomized studies of interventions (ROBINS-I).

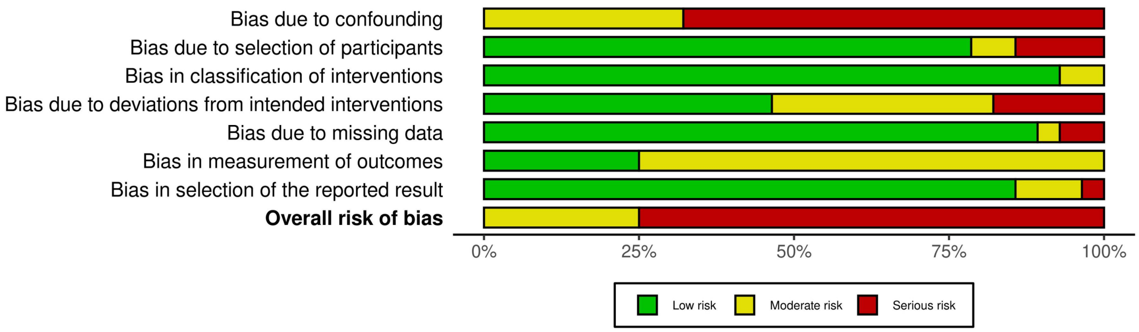

The graph shows the proportion of studies rated as low, moderate, and serious risk for each bias domain according to the Cochrane ROBINS-I tool.

Forest plots of the meta-analysis at different time points of the primary outcome

Supplementary Figure 3. Forest plot of the pooled risk difference (RD) for survival to hospital discharge in ECPR patients treated with versus without therapeutic hypothermia.

(a) RCTs

Study or Subgroup	Events	Total	Events	Total	Weight	Risk Difference M-H, Random, 95% CI
Pang 2016	3	9	2	12	29.8%	0.17 [-0.21, 0.54]
Pan 2020	12	30	10	30	70.2%	0.07 [-0.18, 0.31]
**Total (95% CI)**		**39**		**42**	**100.0%**	**0.10 [-0.11, 0.30]**
Total events	15		12			

Heterogeneity: Tau² = 0.00; Chi² = 0.20, df = 1 (P = 0.66); I² = 0%
Test for overall effect: Z = 0.93 (P = 0.35)

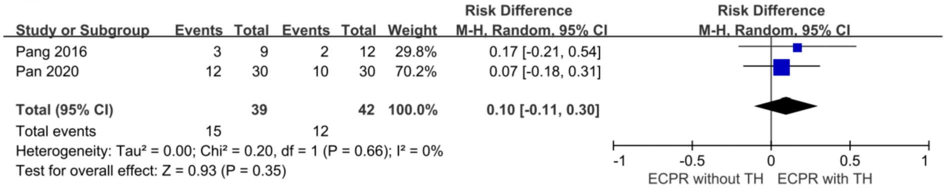

(b) observational studys

Study or Subgroup	Experimental Events	Experimental Total	Control Events	Control Total	Weight	Risk Difference M-H, Random, 95% CI
Dennis 2017	7	15	6	22	3.9%	0.19 [-0.12, 0.51]
Han 2019	8	26	6	74	7.0%	0.23 [0.04, 0.41]
Ijuin 2025	21	293	18	180	12.0%	-0.03 [-0.08, 0.02]
Kagawa 2015	14	48	3	39	8.3%	0.21 [0.06, 0.37]
Kim 2014	5	14	3	38	4.9%	0.28 [0.01, 0.54]
Kim 2018	12	25	35	76	5.9%	0.02 [-0.21, 0.25]
Kim 2024	18	69	16	69	8.6%	0.03 [-0.11, 0.17]
Lee 2026	46	79	51	133	8.9%	0.20 [0.06, 0.34]
Mecklenburg 2021	11	36	10	30	5.8%	-0.03 [-0.25, 0.20]
Pang 2017	7	14	14	65	4.5%	0.28 [0.00, 0.56]
Ryu 2017	16	25	11	17	4.2%	-0.01 [-0.30, 0.29]
Ryu 2019	22	36	75	238	7.7%	0.30 [0.13, 0.47]
Sakamoto 2014	162	231	14	29	6.9%	0.22 [0.03, 0.41]
Yuan 2024	119	385	76	263	11.4%	0.02 [-0.05, 0.09]
**Total (95% CI)**		**1296**		**1273**	**100.0%**	**0.12 [0.05, 0.20]**
Total events	468		338			

Heterogeneity: Tau² = 0.01; Chi² = 42.05, df = 13 (P < 0.0001); I² = 69%
Test for overall effect: Z = 3.27 (P = 0.001)

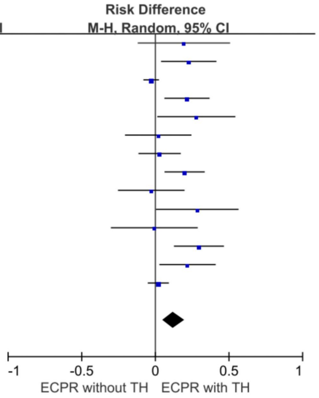

Pooled analysis using random-effects model. Diamond indicates overall effect size; vertical dashed line indicates null effect (RD = 0); horizontal lines indicate 95% confidence intervals for individual studies; square size reflects study weight. TH: therapeutic hypothermia; ECPR: extracorporeal cardiopulmonary resuscitation. (a) represents the results of meta-analysis of RCT studies and (b) represents the results of meta-analysis of observational studies.

Supplementary Figure 4. Forest plot of the pooled risk difference (RD) for neurological outcome at discharge in ECPR patients treated with versus without therapeutic hypothermia.

(a) RCTs

Study or Subgroup	Experimental Events	Experimental Total	Control Events	Control Total	Weight	Risk Difference M-H, Random, 95% CI
Pan 2020	12	30	7	30	64.7%	0.17 [-0.06, 0.40]
Pang 2016	2	9	1	12	35.3%	0.14 [-0.17, 0.45]
**Total (95% CI)**		**39**		**42**	**100.0%**	**0.16 [-0.03, 0.34]**
Total events	14		8			

Heterogeneity: Tau² = 0.00; Chi² = 0.02, df = 1 (P = 0.89); I² = 0%
Test for overall effect: Z = 1.65 (P = 0.10)

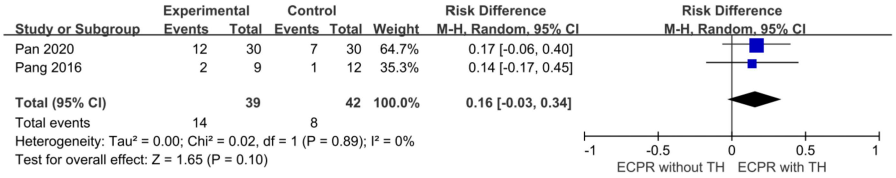

(b) observational studys

Study or Subgroup	Experimental Events	Experimental Total	Control Events	Control Total	Weight	Risk Difference M-H, Random, 95% CI
Al-Kawaz 2022	3	10	8	29	2.4%	0.02 [-0.30, 0.35]
Dennis 2017	7	15	6	22	2.6%	0.19 [-0.12, 0.51]
Han 2019	8	26	6	74	5.5%	0.23 [0.04, 0.41]
Ijuin 2025	43	293	37	180	12.0%	-0.06 [-0.13, 0.01]
Kim 2018	8	25	26	76	4.7%	-0.02 [-0.23, 0.19]
Kim 2024	11	69	10	69	8.8%	0.01 [-0.11, 0.13]
Kobata 2020	5	18	1	4	1.3%	0.03 [-0.44, 0.50]
Lee 2026	23	79	14	133	9.3%	0.19 [0.07, 0.30]
Otani 2020	11	33	13	123	6.2%	0.23 [0.06, 0.40]
Pang 2017	6	14	10	65	3.2%	0.27 [0.00, 0.55]
Ryu 2017	14	25	9	17	2.7%	0.03 [-0.28, 0.34]
Ryu 2019	16	36	62	238	6.2%	0.18 [0.01, 0.36]
Sakamoto 2014	30	231	2	29	10.0%	0.06 [-0.04, 0.16]
Shih 2024	12	55	3	30	7.0%	0.12 [-0.03, 0.27]
Yamada 2021	38	202	8	66	10.4%	0.07 [-0.03, 0.16]
Yukawa 2017	9	50	2	29	7.6%	0.11 [-0.03, 0.25]
**Total (95% CI)**		**1181**		**1184**	**100.0%**	**0.09 [0.04, 0.15]**
Total events	244		217			

Heterogeneity: Tau² = 0.01; Chi² = 29.53, df = 15 (P = 0.01); I² = 49%
Test for overall effect: Z = 3.32 (P = 0.0009)

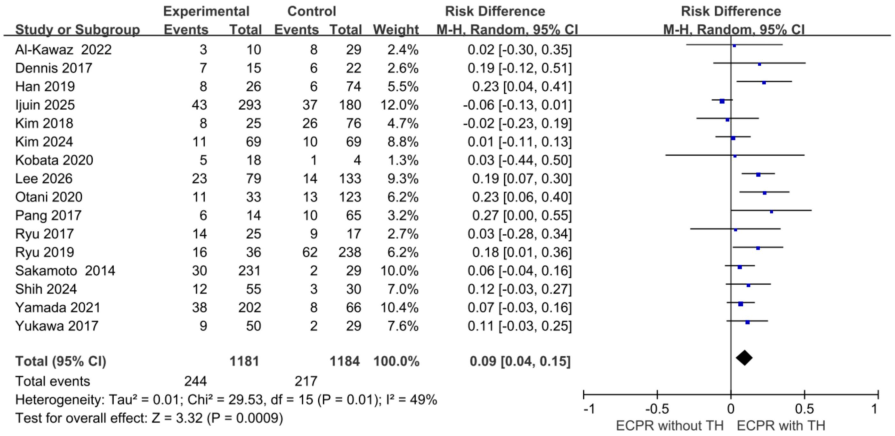

Pooled analysis using random-effects model. Diamond indicates overall effect size; vertical dashed line indicates null effect (RD = 0); horizontal lines indicate 95% confidence intervals for individual studies; square size reflects study weight. TH: therapeutic hypothermia; ECPR: extracorporeal cardiopulmonary resuscitation. (a) represents the results of meta-analysis of RCT studies and (b) represents the results of meta-analysis of observational studies.

Supplemental Figure 5 Forest plot of meta-analysis for survival at 1 month (RR).(Only observational studies were pooled)

	ECPR with TH		ECPR without TH			Risk Ratio
Study or Subgroup	Events	Total	Events	Total	Weight	M-H, Random, 95% CI
Choi 2016	3	6	0	4	5.6%	5.00 [0.33, 76.81]
Kim 2021	10	38	26	146	32.1%	1.48 [0.78, 2.79]
Lee 2016	6	18	4	5	28.1%	0.42 [0.19, 0.92]
Mecklenburg 2021	14	36	14	30	34.2%	0.83 [0.48, 1.46]
**Total (95% CI)**		**98**		**185**	**100.0%**	**0.91 [0.46, 1.82]**
Total events	33		44			

Heterogeneity: Tau² = 0.28; Chi² = 8.22, df = 3 (P = 0.04); I² = 64%
Test for overall effect: Z = 0.26 (P = 0.79)

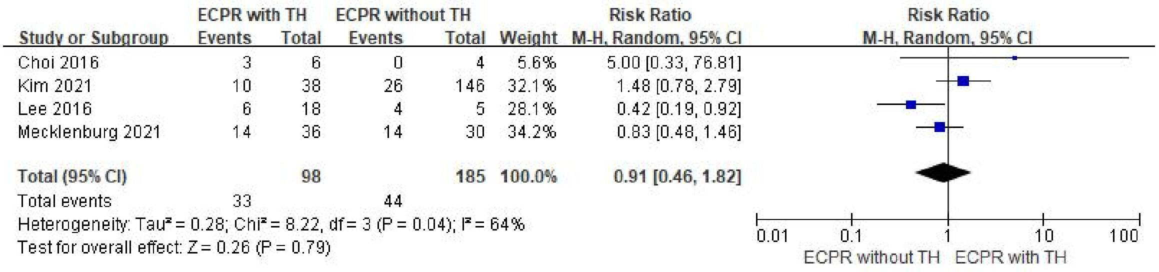

Pooled analysis using random-effects model. Diamond indicates overall effect size; vertical dashed line indicates null effect (RR = 1); horizontal lines indicate 95% confidence intervals for individual studies; square size reflects study weight. TH: therapeutic hypothermia; ECPR: extracorporeal cardiopulmonary resuscitation.

Supplemental Figure 6 Forest plot of meta-analysis for survival at 1 month (RD).(Only observational studies were pooled)

Study or Subgroup	ECPR with TH Events	ECPR with TH Total	ECPR without TH Events	ECPR without TH Total	Weight	Risk Difference M-H, Random, 95% CI
Choi 2016	3	6	0	4	18.2%	0.50 [0.05, 0.95]
Kim 2021	10	38	26	146	33.1%	0.09 [-0.07, 0.24]
Lee 2016	6	18	4	5	20.0%	-0.47 [-0.88, -0.05]
Mecklenburg 2021	14	36	14	30	28.7%	-0.08 [-0.32, 0.16]
**Total (95% CI)**		**98**		**185**	**100.0%**	**0.00 [-0.27, 0.28]**
Total events	33		44			

Heterogeneity: Tau² = 0.05; Chi² = 11.01, df = 3 (P = 0.01); I² = 73%

Test for overall effect: Z = 0.03 (P = 0.98)

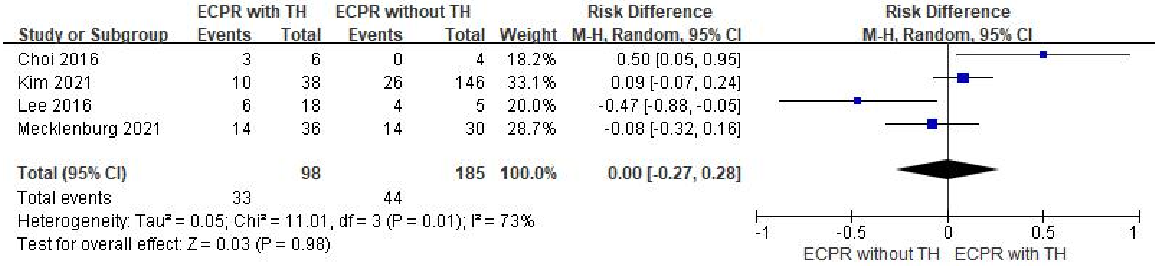

Pooled analysis using random-effects model. Diamond indicates overall effect size; vertical dashed line indicates null effect (RD = 0); horizontal lines indicate 95% confidence intervals for individual studies; square size reflects study weight. TH: therapeutic hypothermia; ECPR: extracorporeal cardiopulmonary resuscitation.

Supplemental Figure 7 Forest plot of meta-analysis for survival at 3 months (RR).(Only observational studies were pooled)

Study or Subgroup	ECPR with TH Events	ECPR with TH Total	ECPR without TH Events	ECPR without TH Total	Weight	Risk Ratio M-H, Random, 95% CI
Maekawa 2013	10	26	7	26	10.0%	1.43 [0.64, 3.17]
Nakashima 2022	259	838	221	653	90.0%	0.91 [0.79, 1.06]
**Total (95% CI)**		**864**		**679**	**100.0%**	**0.95 [0.73, 1.24]**
Total events	269		228			

Heterogeneity: Tau² = 0.01; Chi² = 1.17, df = 1 (P = 0.28); I² = 14%
Test for overall effect: Z = 0.34 (P = 0.73)

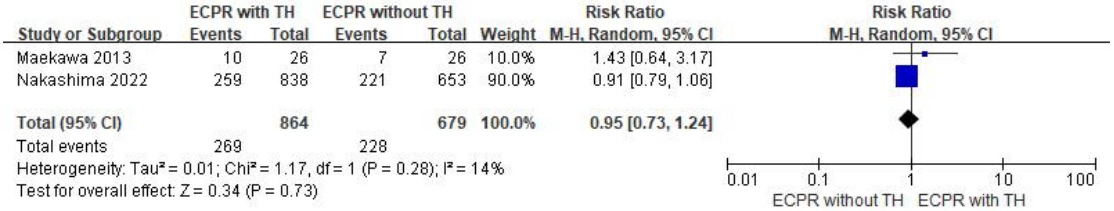

Pooled analysis using random-effects model. Diamond indicates overall effect size; vertical dashed line indicates null effect (RR = 1); horizontal lines indicate 95% confidence intervals for individual studies; square size reflects study weight. TH: therapeutic hypothermia; ECPR: extracorporeal cardiopulmonary resuscitation.

Supplemental Figure 8 Forest plot of meta-analysis for survival at 3 months (RD).(Only observational studies were pooled)

	ECPR with TH		ECPR without TH			Risk Difference
Study or Subgroup	Events	Total	Events	Total	Weight	M-H, Random, 95% CI
Maekawa 2013	10	26	7	26	11.6%	0.12 [-0.14, 0.37]
Nakashima 2022	259	838	221	653	88.4%	-0.03 [-0.08, 0.02]
**Total (95% CI)**		**864**		**679**	**100.0%**	**-0.01 [-0.10, 0.08]**
Total events	269		228			

Heterogeneity: $Tau^2 = 0.00$; $Chi^2 = 1.21$, df = 1 (P = 0.27); $I^2 = 18\%$
Test for overall effect: Z = 0.27 (P = 0.79)

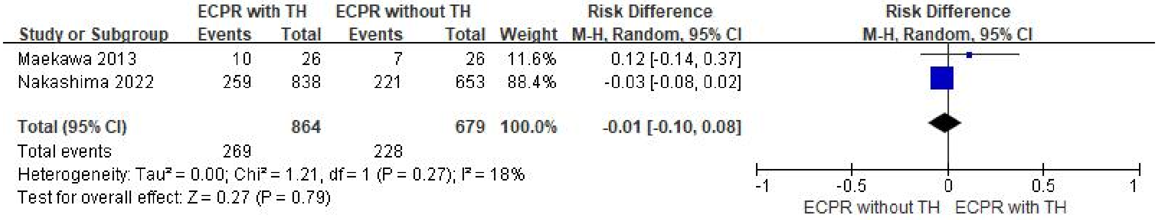

Pooled analysis using random-effects model. Diamond indicates overall effect size; vertical dashed line indicates null effect (RD = 0); horizontal lines indicate 95% confidence intervals for individual studies; square size reflects study weight. TH: therapeutic hypothermia; ECPR: extracorporeal cardiopulmonary resuscitation.

Supplemental Figure 9 Forest plot of meta-analysis for survival at 6 months (RR). (Only RCTs were pooled)

	ECPR with TH		ECPR without TH			Risk Ratio
Study or Subgroup	Events	Total	Events	Total	Weight	M-H, Random, 95% CI
Lewy 2022	80	168	70	166	84.8%	1.13 [0.89, 1.43]
Pang 2016	3	9	1	12	15.2%	4.00 [0.49, 32.39]
**Total (95% CI)**		**177**		**178**	**100.0%**	**1.37 [0.56, 3.35]**
Total events	83		71			

Heterogeneity: Tau² = 0.23; Chi² = 1.40, df = 1 (P = 0.24); I² = 29%
Test for overall effect: Z = 0.69 (P = 0.49)

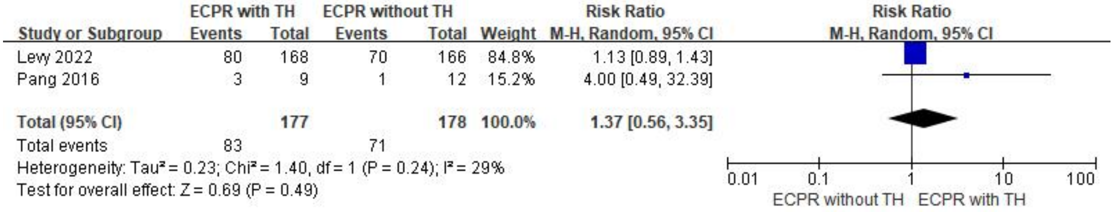

Pooled analysis using random-effects model. Diamond indicates overall effect size; vertical dashed line indicates null effect (RR = 1); horizontal lines indicate 95% confidence intervals for individual studies; square size reflects study weight. TH: therapeutic hypothermia; ECPR: extracorporeal cardiopulmonary resuscitation.

Supplemental Figure 10 Forest plot of meta-analysis for survival at 6 months (RD).(Only RCTs were pooled)

	ECPR with TH		ECPR without TH			Risk Difference
Study or Subgroup	Events	Total	Events	Total	Weight	M-H, Random, 95% CI
Lew 2022	80	168	70	166	86.4%	0.05 [-0.05, 0.16]
Pang 2016	3	9	1	12	13.6%	0.25 [-0.10, 0.60]
**Total (95% CI)**		**177**		**178**	**100.0%**	**0.08 [-0.05, 0.21]**
Total events	83		71			
Heterogeneity: Tau² = 0.00; Chi² = 1.14, df = 1 (P = 0.29); I² = 12%						
Test for overall effect: Z = 1.20 (P = 0.23)						

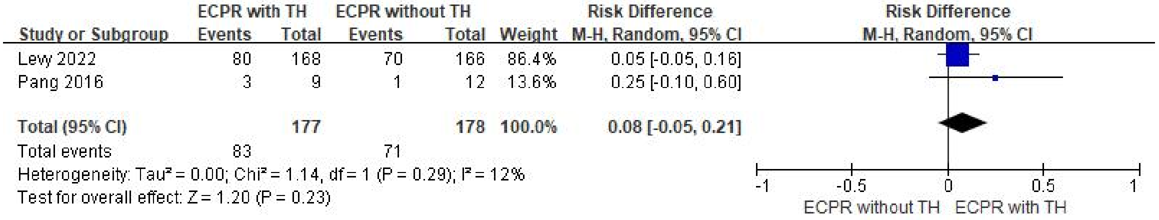

Pooled analysis using random-effects model. Diamond indicates overall effect size; vertical dashed line indicates null effect (RD = 0); horizontal lines indicate 95% confidence intervals for individual studies; square size reflects study weight. TH: therapeutic hypothermia; ECPR: extracorporeal cardiopulmonary resuscitation.

Supplemental Figure 11 Forest plot of meta-analysis for favorable neurological outcome at 1 month (RR).(Only observational studies were pooled)

	ECPR with TH		ECPR without TH			Risk Ratio
Study or Subgroup	Events	Total	Events	Total	Weight	M-H, Random, 95% CI
Choi 2016	3	6	0	4	2.4%	5.00 [0.33, 76.81]
Pöss 2025	4	27	5	34	11.6%	1.01 [0.30, 3.39]
Sakurai 2022	75	471	50	506	77.7%	1.61 [1.15, 2.25]
Taira 2025	3	36	4	227	8.3%	4.73 [1.10, 20.26]
**Total (95% CI)**		**540**		**771**	**100.0%**	**1.71 [1.12, 2.64]**
Total events	85		59			

Heterogeneity: $Tau^2 = 0.03$; $Chi^2 = 3.29$, df = 3 (P = 0.35); $I^2 = 9\%$

Test for overall effect: Z = 2.46 (P = 0.01)

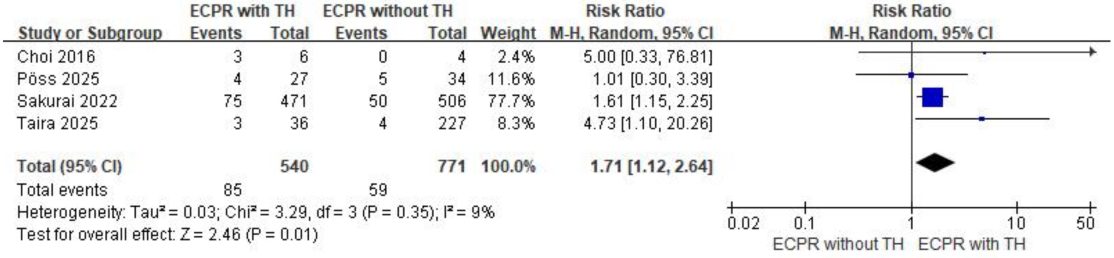

Pooled analysis using random-effects model. Diamond indicates overall effect size; vertical dashed line indicates null effect (RR = 1); horizontal lines indicate 95% confidence intervals for individual studies; square size reflects study weight. TH: therapeutic hypothermia; ECPR: extracorporeal cardiopulmonary resuscitation.

Supplemental Figure 12 Forest plot of meta-analysis for favorable neurological outcome at 1 month (RD).(Only observational studies were pooled)

Study or Subgroup	ECPR with TH Events	ECPR with TH Total	ECPR without TH Events	ECPR without TH Total	Weight	Risk Difference M-H, Random, 95% CI
Choi 2016	3	6	0	4	1.7%	0.50 [0.05, 0.95]
Pöss 2025	4	27	5	34	9.7%	0.00 [-0.18, 0.18]
Sakurai 2022	75	471	50	506	60.5%	0.06 [0.02, 0.10]
Taira 2025	3	36	4	227	28.1%	0.07 [-0.03, 0.16]
**Total (95% CI)**		**540**		**771**	**100.0%**	**0.06 [0.00, 0.12]**
Total events	85		59			

Heterogeneity: Tau² = 0.00; Chi² = 4.03, df = 3 (P = 0.26); I² = 25%
Test for overall effect: Z = 2.10 (P = 0.04)

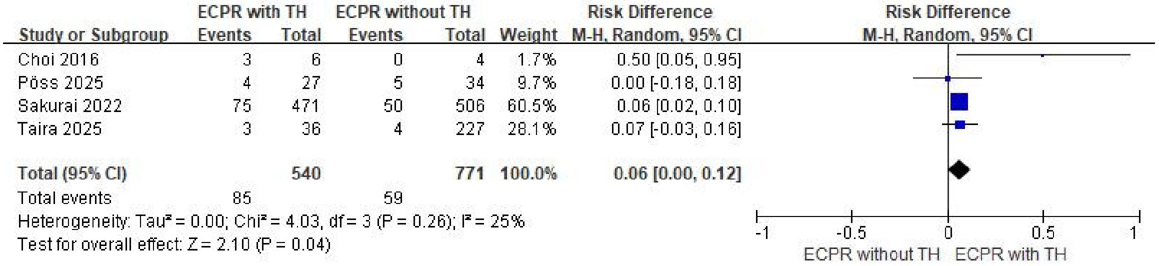

Pooled analysis using random-effects model. Diamond indicates overall effect size; vertical dashed line indicates null effect (RD = 0); horizontal lines indicate 95% confidence intervals for individual studies; square size reflects study weight. TH: therapeutic hypothermia; ECPR: extracorporeal cardiopulmonary resuscitation.

Supplemental Figure 13 Forest plot of meta-analysis for favorable neurological outcome at 3 months (RR).(Only observational studies were pooled)

Study or Subgroup	ECPR with TH Events	ECPR with TH Total	ECPR without TH Events	ECPR without TH Total	Weight	Risk Ratio M-H, Random, 95% CI
Kagawa 2015	12	48	3	39	49.2%	3.25 [0.99, 10.71]
Kim 2014	5	14	3	38	41.8%	4.52 [1.24, 16.49]
Maekawa 2013	8	26	0	26	8.9%	17.00 [1.03, 280.07]
**Total (95% CI)**		**88**		**103**	**100.0%**	**4.33 [1.87, 9.99]**
Total events	25		6			

Heterogeneity: Tau² = 0.00; Chi² = 1.23, df = 2 (P = 0.54); I² = 0%
Test for overall effect: Z = 3.43 (P = 0.0006)

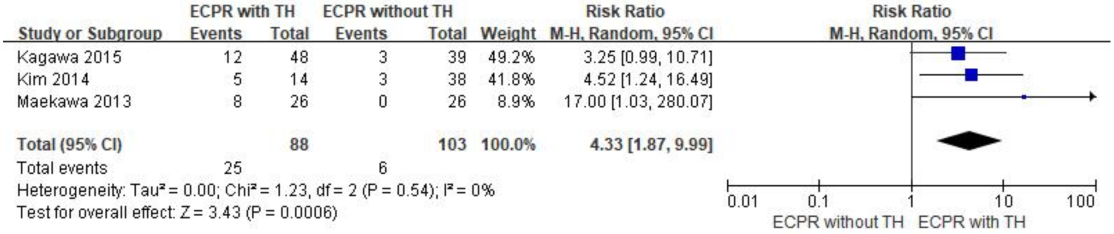

Pooled analysis using random-effects model. Diamond indicates overall effect size; vertical dashed line indicates null effect (RR = 1); horizontal lines indicate 95% confidence intervals for individual studies; square size reflects study weight. TH: therapeutic hypothermia; ECPR: extracorporeal cardiopulmonary resuscitation.

Supplemental Figure 14 Forest plot of meta-analysis for favorable neurological outcome at 3 months (RD).(Only observational studies were pooled)

	ECPR with TH		ECPR without TH			Risk Difference
Study or Subgroup	Events	Total	Events	Total	Weight	M-H, Random, 95% CI
Kagawa 2015	12	48	3	39	50.7%	0.17 [0.02, 0.32]
Kim 2014	5	14	3	38	15.8%	0.28 [0.01, 0.54]
Maekawa 2013	8	26	0	26	33.5%	0.31 [0.13, 0.49]
**Total (95% CI)**		**88**		**103**	**100.0%**	**0.23 [0.13, 0.34]**
Total events	25		6			

Heterogeneity: Tau² = 0.00; Chi² = 1.38, df = 2 (P = 0.50); I² = 0%
Test for overall effect: Z = 4.36 (P < 0.0001)

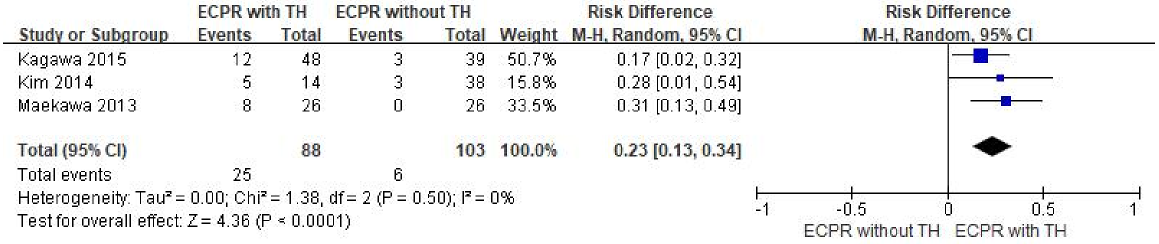

Pooled analysis using random-effects model. Diamond indicates overall effect size; vertical dashed line indicates null effect (RD = 0); horizontal lines indicate 95% confidence intervals for individual studies; square size reflects study weight. TH: therapeutic hypothermia; ECPR: extracorporeal cardiopulmonary resuscitation.

Forest plots of the meta-analysis of different complications in the secondary outcomes

Supplemental Figure 15 Forest plot of meta-analysis for hemorrhage complications in ECPR patients treated with versus without therapeutic hypothermia.(Only RCTs were pooled)

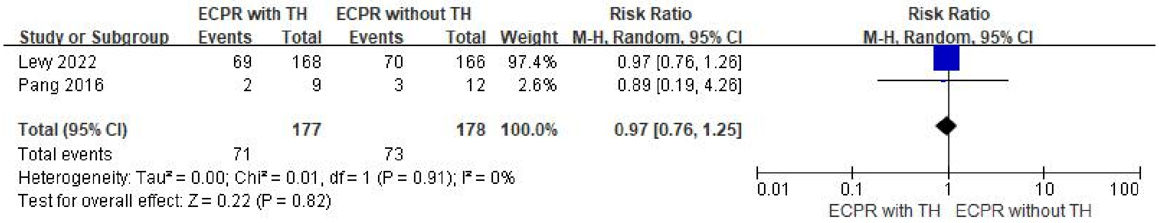

Pooled analysis using a random-effects model. Diamond indicates overall effect size; vertical dashed line indicates null effect (RR = 1); horizontal lines indicate 95% confidence intervals for individual studies; square size reflects study weight. TH: therapeutic hypothermia; ECPR: extracorporeal cardiopulmonary resuscitation.

Supplemental Figure 16 Forest plot of meta-analysis for hemorrhage complications in ECPR patients treated with versus without therapeutic hypothermia.(Only observational studies were pooled)

	ECPR with TH		ECPR without TH			Risk Ratio
Study or Subgroup	Events	Total	Events	Total	Weight	M-H, Random, 95% CI
Kim 2018	15	25	37	76	25.0%	1.23 [0.83, 1.83]
Lee 2026	9	79	22	133	11.9%	0.69 [0.33, 1.42]
Mecklenburg 2021	14	36	17	30	18.8%	0.69 [0.41, 1.15]
Nakashima 2022	253	849	161	662	40.0%	1.23 [1.03, 1.45]
Pang 2017	2	14	18	65	4.3%	0.52 [0.13, 1.97]
**Total (95% CI)**		**1003**		**966**	**100.0%**	**0.99 [0.74, 1.32]**
Total events	293		255			

Heterogeneity: $Tau^2 = 0.05$; $Chi^2 = 7.77$, df = 4 (P = 0.10); $I^2 = 49\%$
Test for overall effect: Z = 0.07 (P = 0.94)

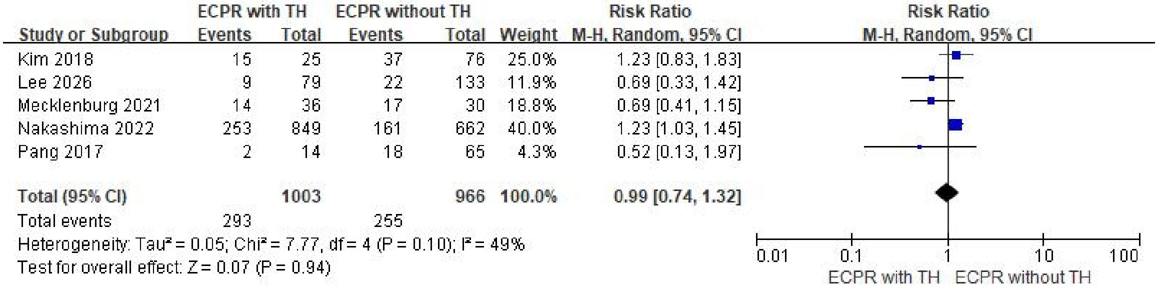

Pooled analysis using a random-effects model. Diamond indicates overall effect size; vertical dashed line indicates null effect (RR = 1); horizontal lines indicate 95% confidence intervals for individual studies; square size reflects study weight. TH: therapeutic hypothermia; ECPR: extracorporeal cardiopulmonary resuscitation.

Supplemental Figure 17 Forest plot of meta-analysis for infection complications in ECPR patients treated with versus without therapeutic hypothermia.(Only RCTs were pooled)

	ECPR with TH		ECPR without TH			Risk Ratio
Study or Subgroup	Events	Total	Events	Total	Weight	M-H, Random, 95% CI
Lew 2022	87	168	86	166	78.3%	1.00 [0.81, 1.23]
Pan 2020	10	30	11	30	18.9%	0.91 [0.46, 1.81]
Pang 2016	1	9	6	12	2.8%	0.22 [0.03, 1.53]
**Total (95% CI)**		**207**		**208**	**100.0%**	**0.94 [0.68, 1.31]**
Total events	98		103			

Heterogeneity: Tau² = 0.02; Chi² = 2.41, df = 2 (P = 0.30); I² = 17%

Test for overall effect: Z = 0.36 (P = 0.72)

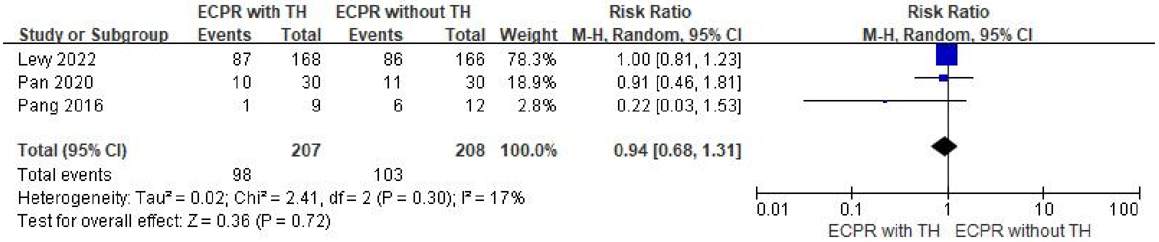

Pooled analysis using a random-effects model. Diamond indicates overall effect size; vertical dashed line indicates null effect (RR = 1); horizontal lines indicate 95% confidence intervals for individual studies; square size reflects study weight. TH: therapeutic hypothermia; ECPR: extracorporeal cardiopulmonary resuscitation.

Supplemental Figure 18 Forest plot of meta-analysis for infection complications in ECPR patients treated with versus without therapeutic hypothermia.(Only observational studies were pooled)

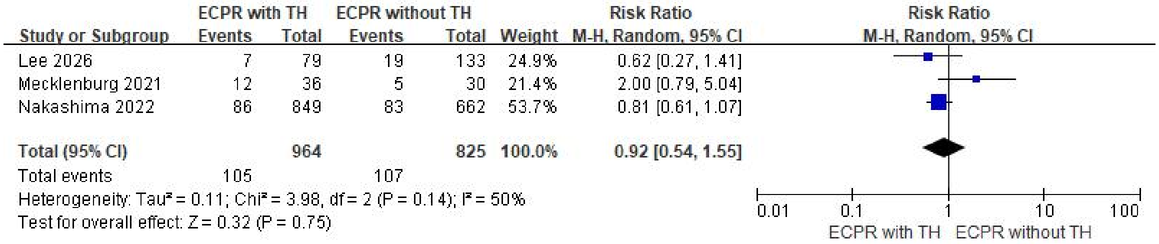

Pooled analysis using a random-effects model. Diamond indicates overall effect size; vertical dashed line indicates null effect (RR = 1); horizontal lines indicate 95% confidence intervals for individual studies; square size reflects study weight. TH: therapeutic hypothermia; ECPR: extracorporeal cardiopulmonary resuscitation.

Supplemental Figure 19 Forest plot of meta-analysis for limb ischaemia complications in ECPR patients treated with versus without therapeutic hypothermia.(Only RCTs were pooled)

	ECPR with TH		ECPR without TH			Risk Ratio
Study or Subgroup	Events	Total	Events	Total	Weight	M-H, Random, 95% CI
Pan 2020	9	30	7	30	74.8%	1.29 [0.55, 3.00]
Pang 2016	2	9	4	12	25.2%	0.67 [0.15, 2.87]
**Total (95% CI)**		**39**		**42**	**100.0%**	**1.09 [0.52, 2.27]**
Total events	11		11			

Heterogeneity: $Tau^2 = 0.00$; $Chi^2 = 0.58$, df = 1 (P = 0.45); $I^2 = 0\%$

Test for overall effect: Z = 0.23 (P = 0.82)

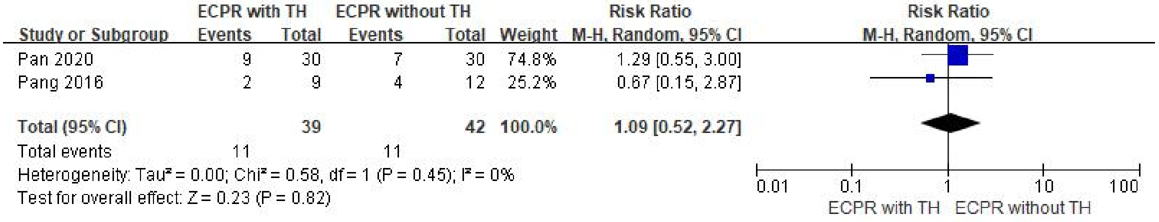

Pooled analysis using a random-effects model. Diamond indicates overall effect size; vertical dashed line indicates null effect (RR = 1); horizontal lines indicate 95% confidence intervals for individual studies; square size reflects study weight. TH: therapeutic hypothermia; ECPR: extracorporeal cardiopulmonary resuscitation.

Supplemental Figure 20 Forest plot of meta-analysis for limb ischaemia complications in ECPR patients treated with versus without therapeutic hypothermia.(Only observational studies were pooled)

Study or Subgroup	ECPR with TH Events	ECPR with TH Total	ECPR without TH Events	ECPR without TH Total	Weight	Risk Ratio M-H, Random, 95% CI
Kim 2018	3	25	7	76	7.3%	1.30 [0.36, 4.66]
Nakashima 2022	60	849	46	662	86.3%	1.02 [0.70, 1.47]
Pang 2017	2	14	14	65	6.4%	0.66 [0.17, 2.60]
**Total (95% CI)**		**888**		**803**	**100.0%**	**1.01 [0.71, 1.42]**
Total events	65		67			

Heterogeneity: Tau² = 0.00; Chi² = 0.52, df = 2 (P = 0.77); I² = 0%
Test for overall effect: Z = 0.04 (P = 0.96)

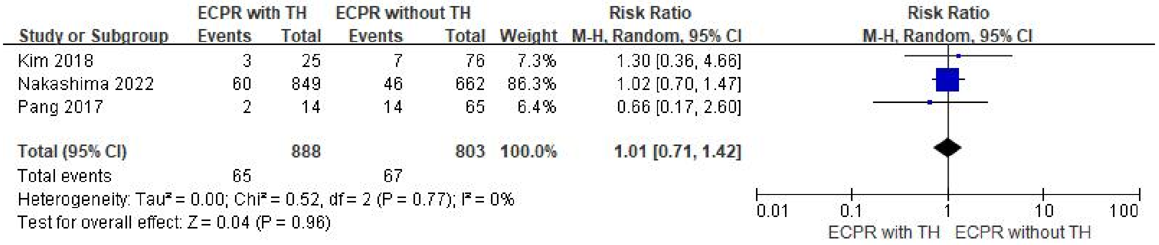

Pooled analysis using a random-effects model. Diamond indicates overall effect size; vertical dashed line indicates null effect (RR = 1); horizontal lines indicate 95% confidence intervals for individual studies; square size reflects study weight. TH: therapeutic hypothermia; ECPR: extracorporeal cardiopulmonary resuscitation.

Supplemental Figure 21 Forest plot of meta-analysis for acute kidney injury complications in ECPR patients treated with versus without therapeutic hypothermia.(Only RCTs were pooled)

	ECPR with TH		ECPR without TH			Risk Ratio
Study or Subgroup	Events	Total	Events	Total	Weight	M-H, Random, 95% CI
Pan 2020	15	30	14	30	23.1%	1.07 [0.63, 1.81]
Pang 2016	8	9	11	12	76.9%	0.97 [0.73, 1.29]
**Total (95% CI)**		**39**		**42**	**100.0%**	**0.99 [0.77, 1.28]**
Total events	23		25			

Heterogeneity: Tau² = 0.00; Chi² = 0.19, df = 1 (P = 0.66); I² = 0%
Test for overall effect: Z = 0.06 (P = 0.95)

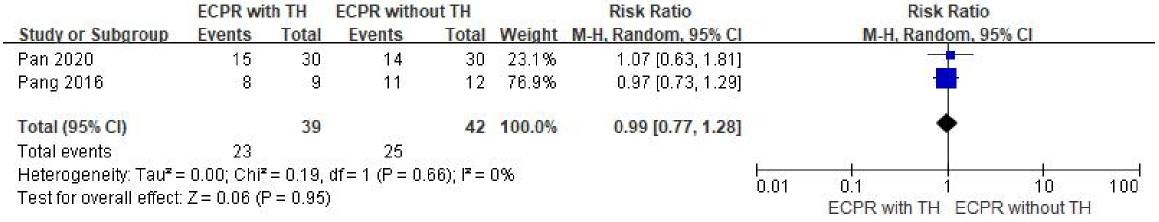

Pooled analysis using a random-effects model. Diamond indicates overall effect size; vertical dashed line indicates null effect (RR = 1); horizontal lines indicate 95% confidence intervals for individual studies; square size reflects study weight. TH: therapeutic hypothermia; ECPR: extracorporeal cardiopulmonary resuscitation.

Supplemental Figure 22 Forest plot of meta-analysis for acute kidney injury complications in ECPR patients treated with versus without therapeutic hypothermia.(Only observational studies were pooled)

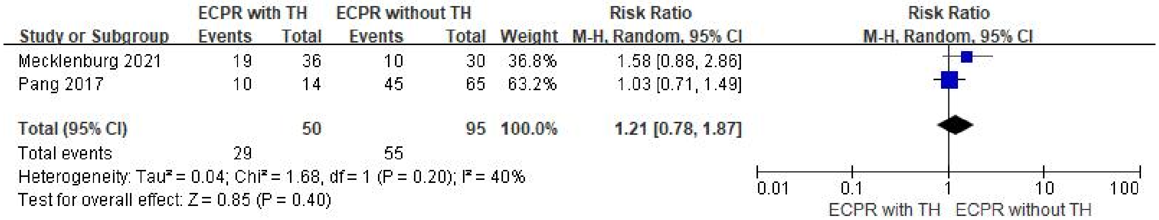

Pooled analysis using a random-effects model. Diamond indicates overall effect size; vertical dashed line indicates null effect (RR = 1); horizontal lines indicate 95% confidence intervals for individual studies; square size reflects study weight. TH: therapeutic hypothermia; ECPR: extracorporeal cardiopulmonary resuscitation.

Supplemental Figure 23 Forest plot of meta-analysis for liver injury complications in ECPR patients treated with versus without therapeutic hypothermia.(Only observational studies were pooled)

	ECPR with TH		ECPR without TH			Risk Ratio
Study or Subgroup	Events	Total	Events	Total	Weight	M-H, Random, 95% CI
Mecklenburg 2021	27	36	27	30	95.8%	0.83 [0.67, 1.04]
Pang 2017	3	14	19	65	4.2%	0.73 [0.25, 2.14]
**Total (95% CI)**		**50**		**95**	**100.0%**	**0.83 [0.67, 1.03]**
Total events	30		46			
Heterogeneity: Tau² = 0.00; Chi² = 0.08, df = 1 (P = 0.78); I² = 0%						
Test for overall effect: Z = 1.68 (P = 0.09)						

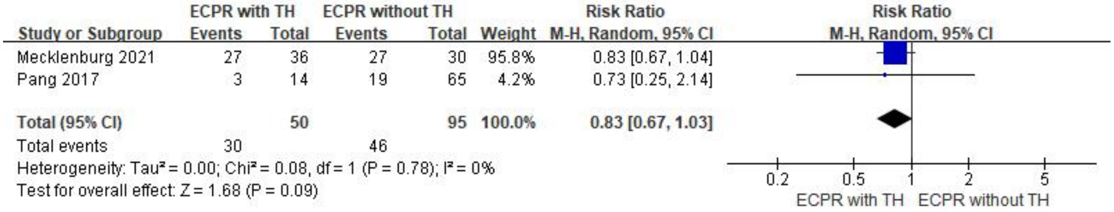

Pooled analysis using a random-effects model. Diamond indicates overall effect size; vertical dashed line indicates null effect (RR = 1); horizontal lines indicate 95% confidence intervals for individual studies; square size reflects study weight. TH: therapeutic hypothermia; ECPR: extracorporeal cardiopulmonary resuscitation.

Subgroup analysis

Supplemental Figure 24 Meta-analysis of the effect of ECPR with TH on survival to discharge: Subgroup analysis based on patient population .(Only observational studies were pooled)

	ECPR with TH		ECPR without TH			Risk Ratio
Study or Subgroup	Events	Total	Events	Total	Weight	M-H, Random, 95% CI
**1.33.1 OHCA and IHCA**						
Dennis 2017	7	15	6	22	4.3%	1.71 [0.72, 4.09]
Han 2019	8	26	6	74	3.7%	3.79 [1.45, 9.91]
Kagawa 2015	14	48	3	39	2.7%	3.79 [1.17, 12.26]
Kim 2018	12	25	35	76	8.5%	1.04 [0.65, 1.68]
Mecklenburg 2021	11	36	10	30	5.6%	0.92 [0.45, 1.86]
Pang 2017	7	14	14	65	5.7%	2.32 [1.15, 4.67]
Ryu 2017	16	25	11	17	8.7%	0.99 [0.63, 1.56]
Ryu 2019	22	36	75	238	11.0%	1.94 [1.41, 2.67]
Yuan 2024	119	385	76	263	12.3%	1.07 [0.84, 1.36]
**Subtotal (95% CI)**		**610**		**824**	**62.5%**	**1.48 [1.10, 2.00]**
Total events	216		236			
Heterogeneity: Tau² = 0.11; Chi² = 22.56, df = 8 (P = 0.004); I² = 65%						
Test for overall effect: Z = 2.57 (P = 0.01)						
**1.33.2 Pure OHCA**						
Ijuin 2025	21	293	18	180	6.8%	0.72 [0.39, 1.31]
Kim 2014	5	14	3	38	2.3%	4.52 [1.24, 16.49]
Kim 2024	18	69	16	69	7.0%	1.13 [0.63, 2.02]
Lee 2026	46	79	51	133	11.6%	1.52 [1.14, 2.02]
Sakamoto 2014	162	231	14	29	9.9%	1.45 [0.99, 2.14]
**Subtotal (95% CI)**		**686**		**449**	**37.5%**	**1.33 [0.94, 1.86]**
Total events	252		102			
Heterogeneity: Tau² = 0.08; Chi² = 8.92, df = 4 (P = 0.06); I² = 55%						
Test for overall effect: Z = 1.61 (P = 0.11)						
**Total (95% CI)**		**1296**		**1273**	**100.0%**	**1.41 [1.14, 1.74]**
Total events	468		338			
Heterogeneity: Tau² = 0.08; Chi² = 31.49, df = 13 (P = 0.003); I² = 59%						
Test for overall effect: Z = 3.12 (P = 0.002)						
Test for subgroup differences: Chi² = 0.23, df = 1 (P = 0.63), I² = 0%						

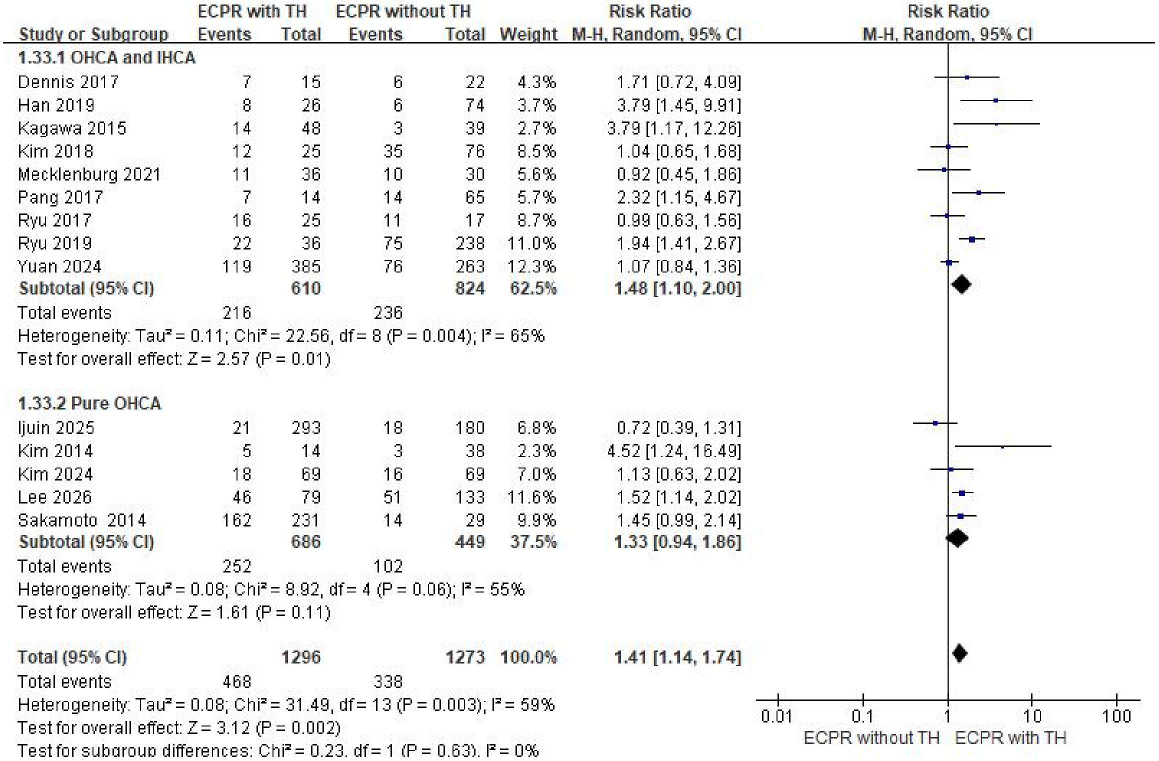

Supplemental Figure 25 Meta-analysis of the effect of ECPR with TH on favorable neurological outcome: Subgroup analysis based on patient population.(Only observational studies were pooled)

Study or Subgroup	ECPR with TH Events	ECPR with TH Total	ECPR without TH Events	ECPR without TH Total	Weight	Risk Ratio M-H, Random, 95% CI
**1.35.1 OHCA and IHCA**						
Al-Kawaz 2022	3	10	8	29	4.4%	1.09 [0.36, 3.32]
Dennis 2017	7	15	6	22	5.9%	1.71 [0.72, 4.09]
Han 2019	8	26	6	74	5.3%	3.79 [1.45, 9.91]
Kim 2018	8	25	26	76	7.8%	0.94 [0.49, 1.79]
Kobata 2020	5	18	1	4	2.0%	1.11 [0.17, 7.09]
Pang 2017	6	14	10	65	6.2%	2.79 [1.21, 6.40]
Ryu 2017	14	25	9	17	8.6%	1.06 [0.60, 1.87]
Ryu 2019	16	36	62	238	10.1%	1.71 [1.12, 2.61]
**Subtotal (95% CI)**		**169**		**525**	**50.2%**	**1.54 [1.12, 2.13]**
Total events	67		128			
Heterogeneity: Tau² = 0.06; Chi² = 10.05, df = 7 (P = 0.19); I² = 30%						
Test for overall effect: Z = 2.63 (P = 0.009)						
**1.35.2 Pure OHCA**						
Ijuin 2025	43	293	37	180	10.4%	0.71 [0.48, 1.06]
Kim 2024	11	69	10	69	6.5%	1.10 [0.50, 2.42]
Lee 2026	23	79	14	133	8.2%	2.77 [1.51, 5.06]
Otani 2020	11	33	13	123	7.3%	3.15 [1.56, 6.38]
Sakamoto 2014	30	231	2	29	3.2%	1.88 [0.47, 7.47]
Shih 2024	12	55	3	30	4.0%	2.18 [0.67, 7.13]
Yamada 2021	38	202	8	66	7.2%	1.55 [0.76, 3.16]
Yukawa 2017	9	50	2	29	3.0%	2.61 [0.60, 11.26]
**Subtotal (95% CI)**		**1012**		**659**	**49.8%**	**1.70 [1.03, 2.80]**
Total events	177		89			
Heterogeneity: Tau² = 0.32; Chi² = 22.82, df = 7 (P = 0.002); I² = 69%						
Test for overall effect: Z = 2.09 (P = 0.04)						
**Total (95% CI)**		**1181**		**1184**	**100.0%**	**1.61 [1.21, 2.14]**
Total events	244		217			
Heterogeneity: Tau² = 0.16; Chi² = 33.15, df = 15 (P = 0.004); I² = 55%						
Test for overall effect: Z = 3.26 (P = 0.001)						
Test for subgroup differences: Chi² = 0.11, df = 1 (P = 0.75), I² = 0%						

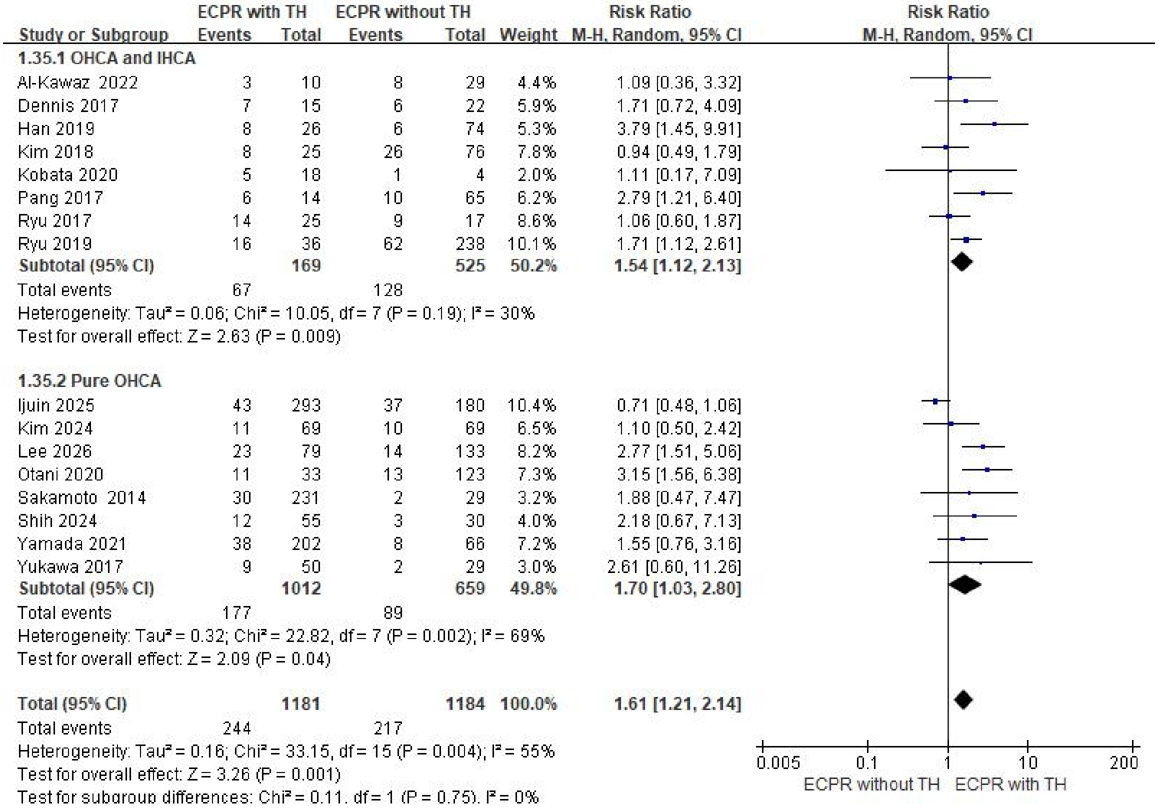

Sensitivity analysis: fixed effects model

Supplemental Figure 26 Forest plot of meta-analysis for survival at discharge.

(a) RCTs

Study or Subgroup	Events	Total	Events	Total	Weight	Risk Ratio M-H, Fixed, 95% CI
Pang 2016	3	9	2	12	14.6%	2.00 [0.42, 9.58]
Pan 2020	12	30	10	30	85.4%	1.20 [0.61, 2.34]
**Total (95% CI)**		**39**		**42**	**100.0%**	**1.32 [0.71, 2.43]**
Total events	15		12			
Heterogeneity: Chi² = 0.35, df = 1 (P = 0.56); I² = 0%						
Test for overall effect: Z = 0.88 (P = 0.38)						

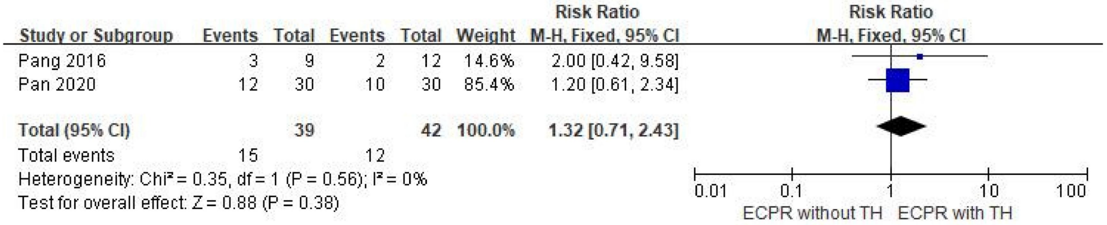

(b) observational studys

Study or Subgroup	Experimental Events	Experimental Total	Control Events	Control Total	Weight	Risk Ratio M-H, Fixed, 95% CI
Dennis 2017	7	15	6	22	1.8%	1.71 [0.72, 4.09]
Han 2019	8	26	6	74	1.2%	3.79 [1.45, 9.91]
Ijuin 2025	21	293	18	180	8.2%	0.72 [0.39, 1.31]
Kagawa 2015	14	48	3	39	1.2%	3.79 [1.17, 12.26]
Kim 2014	5	14	3	38	0.6%	4.52 [1.24, 16.49]
Kim 2018	12	25	35	76	6.4%	1.04 [0.65, 1.68]
Kim 2024	18	69	16	69	5.9%	1.13 [0.63, 2.02]
Lee 2026	46	79	51	133	14.1%	1.52 [1.14, 2.02]
Mecklenburg 2021	11	36	10	30	4.0%	0.92 [0.45, 1.86]
Pang 2017	7	14	14	65	1.8%	2.32 [1.15, 4.67]
Ryu 2017	16	25	11	17	4.8%	0.99 [0.63, 1.56]
Ryu 2019	22	36	75	238	7.3%	1.94 [1.41, 2.67]
Sakamoto 2014	162	231	14	29	9.2%	1.45 [0.99, 2.14]
Yuan 2024	119	385	76	263	33.4%	1.07 [0.84, 1.36]
**Total (95% CI)**		**1296**		**1273**	**100.0%**	**1.31 [1.16, 1.49]**
Total events	468		338			
Heterogeneity: Chi² = 31.49, df = 13 (P = 0.003); I² = 59%						
Test for overall effect: Z = 4.34 (P < 0.0001)						

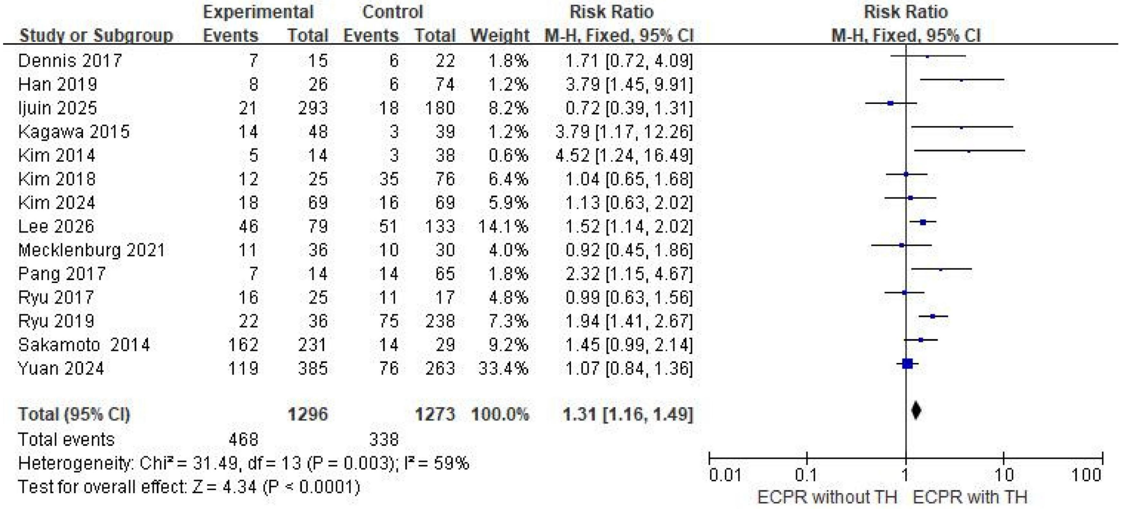

Pooled analysis using fixed-effects model. Diamond indicates overall effect size; vertical dashed line indicates null effect (RR = 1); horizontal lines indicate 95% confidence intervals for individual studies; square size reflects study weight. TH: therapeutic hypothermia; ECPR: extracorporeal cardiopulmonary resuscitation. (a) represents the results of meta-analysis of RCT studies and (b) represents the results of meta-analysis of observational studies.

Supplemental Figure 27 Forest plot of meta-analysis for survival at 1 month. (Only observational studies were pooled)

Study or Subgroup	ECPR with TH Events	ECPR with TH Total	ECPR without TH Events	ECPR without TH Total	Weight	Risk Ratio M-H, Fixed, 95% CI
Choi 2016	3	6	0	4	1.8%	5.00 [0.33, 76.81]
Kim 2021	10	38	26	146	32.7%	1.48 [0.78, 2.79]
Lee 2016	6	18	4	5	19.1%	0.42 [0.19, 0.92]
Mecklenburg 2021	14	36	14	30	46.5%	0.83 [0.48, 1.46]
**Total (95% CI)**		**98**		**185**	**100.0%**	**1.04 [0.72, 1.50]**
Total events	33		44			

Heterogeneity: $Chi^2 = 8.22$, $df = 3$ ($P = 0.04$); $I^2 = 64\%$
Test for overall effect: $Z = 0.20$ ($P = 0.84$)

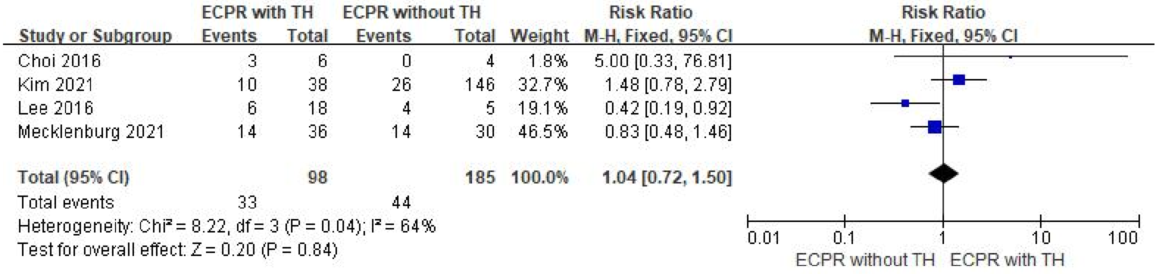

Pooled analysis using fixed-effects model. Diamond indicates overall effect size; vertical dashed line indicates null effect (RR = 1); horizontal lines indicate 95% confidence intervals for individual studies; square size reflects study weight. TH: therapeutic hypothermia; ECPR: extracorporeal cardiopulmonary resuscitation.

Supplemental Figure 28 Forest plot of meta-analysis for survival at 3 months. (Only observational studies were pooled)

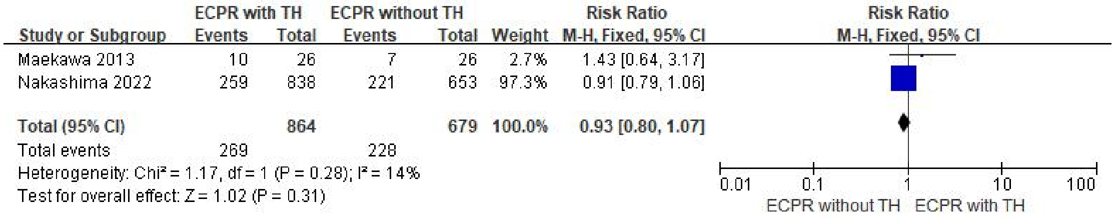

Pooled analysis using fixed-effects model. Diamond indicates overall effect size; vertical dashed line indicates null effect (RR = 1); horizontal lines indicate 95% confidence intervals for individual studies; square size reflects study weight. TH: therapeutic hypothermia; ECPR: extracorporeal cardiopulmonary resuscitation.

Supplemental Figure 29 Forest plot of meta-analysis for survival at 6 months. (Only RCTs were pooled)

	ECPR with TH		ECPR without TH			Risk Ratio
Study or Subgroup	Events	Total	Events	Total	Weight	M-H, Fixed, 95% CI
Lewy 2022	80	168	70	166	98.8%	1.13 [0.89, 1.43]
Pang 2016	3	9	1	12	1.2%	4.00 [0.49, 32.39]
**Total (95% CI)**		**177**		**178**	**100.0%**	**1.16 [0.92, 1.47]**
Total events	83		71			
Heterogeneity: Chi² = 1.40, df = 1 (P = 0.24); I² = 29%						
Test for overall effect: Z = 1.26 (P = 0.21)						

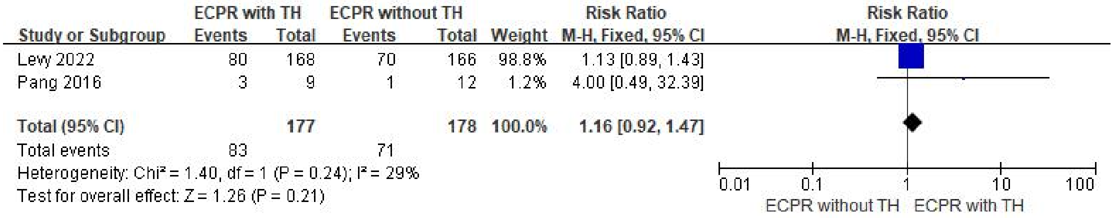

Pooled analysis using fixed-effects model. Diamond indicates overall effect size; vertical dashed line indicates null effect (RR = 1); horizontal lines indicate 95% confidence intervals for individual studies; square size reflects study weight. TH: therapeutic hypothermia; ECPR: extracorporeal cardiopulmonary resuscitation.

Supplemental Figure 30 Forest plot of meta-analysis for favorable neurological outcome at discharge.

(a) RCTs

Study or Subgroup	Experimental Events	Experimental Total	Control Events	Control Total	Weight	Risk Ratio M-H, Fixed, 95% CI
Pan 2020	12	30	7	30	89.1%	1.71 [0.78, 3.75]
Pang 2016	2	9	1	12	10.9%	2.67 [0.28, 25.04]
**Total (95% CI)**		**39**		**42**	**100.0%**	**1.82 [0.87, 3.81]**
Total events	14		8			

Heterogeneity: Chi² = 0.13, df = 1 (P = 0.71); I² = 0%

Test for overall effect: Z = 1.59 (P = 0.11)

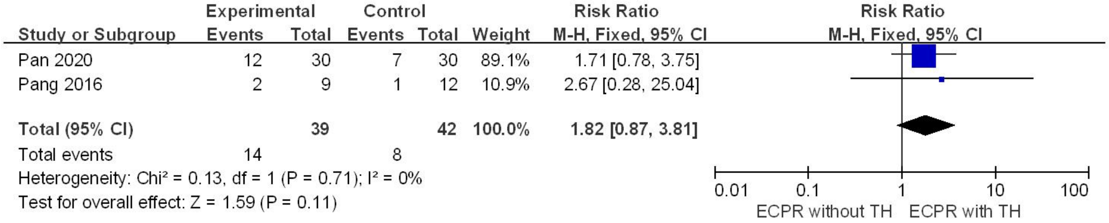

(b) observational studys

Study or Subgroup	Experimental Events	Experimental Total	Control Events	Control Total	Weight	Risk Ratio M-H, Fixed, 95% CI
Al-Kawaz 2022	3	10	8	29	2.7%	1.09 [0.36, 3.32]
Dennis 2017	7	15	6	22	3.2%	1.71 [0.72, 4.09]
Han 2019	8	26	6	74	2.1%	3.79 [1.45, 9.91]
Ijuin 2025	43	293	37	180	30.4%	0.71 [0.48, 1.06]
Kim 2018	8	25	26	76	8.5%	0.94 [0.49, 1.79]
Kim 2024	11	69	10	69	6.6%	1.10 [0.50, 2.42]
Kobata 2020	5	18	1	4	1.1%	1.11 [0.17, 7.09]
Lee 2026	23	79	14	133	6.9%	2.77 [1.51, 5.06]
Otani 2020	11	33	13	123	3.6%	3.15 [1.56, 6.38]
Pang 2017	6	14	10	65	2.3%	2.79 [1.21, 6.40]
Ryu 2017	14	25	9	17	7.1%	1.06 [0.60, 1.87]
Ryu 2019	16	36	62	238	10.8%	1.71 [1.12, 2.61]
Sakamoto 2014	30	231	2	29	2.4%	1.88 [0.47, 7.47]
Shih 2024	12	55	3	30	2.6%	2.18 [0.67, 7.13]
Yamada 2021	38	202	8	66	8.0%	1.55 [0.76, 3.16]
Yukawa 2017	9	50	2	29	1.7%	2.61 [0.60, 11.26]
**Total (95% CI)**		**1181**		**1184**	**100.0%**	**1.44 [1.21, 1.72]**
Total events	244		217			

Heterogeneity: Chi² = 33.15, df = 15 (P = 0.004); I² = 55%

Test for overall effect: Z = 4.10 (P < 0.0001)

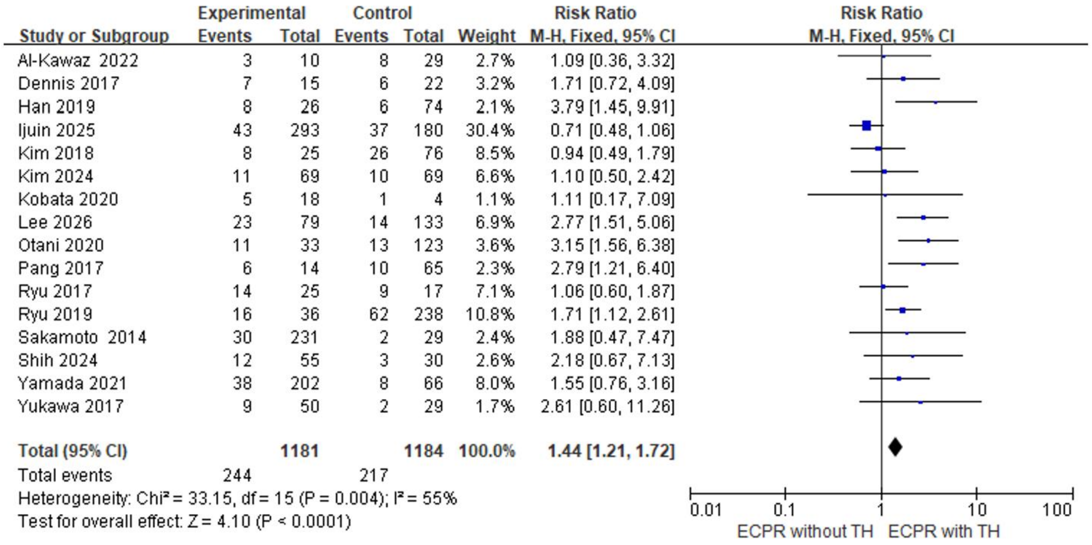

Pooled analysis using fixed-effects model. Diamond indicates overall effect size; vertical dashed line indicates null effect (RR = 1); horizontal lines indicate 95% confidence intervals for individual studies; square size reflects study weight. TH: therapeutic hypothermia; ECPR: extracorporeal cardiopulmonary resuscitation. (a) represents the results of meta-analysis of RCT studies and (b) represents the results of meta-analysis of observational studies.

Supplemental Figure 31 Forest plot of meta-analysis for favorable neurological outcome at 1 month. (Only observational studies were pooled)

	ECPR with TH		ECPR without TH			Risk Ratio
Study or Subgroup	Events	Total	Events	Total	Weight	M-H, Fixed, 95% CI
Choi 2016	3	6	0	4	1.1%	5.00 [0.33, 76.81]
Pöss 2025	4	27	5	34	8.1%	1.01 [0.30, 3.39]
Sakurai 2022	75	471	50	506	88.8%	1.61 [1.15, 2.25]
Taira 2025	3	36	4	227	2.0%	4.73 [1.10, 20.26]
**Total (95% CI)**		**540**		**771**	**100.0%**	**1.66 [1.22, 2.27]**
Total events	85		59			

Heterogeneity: Chi² = 3.29, df = 3 (P = 0.35); I² = 9%
Test for overall effect: Z = 3.20 (P = 0.001)

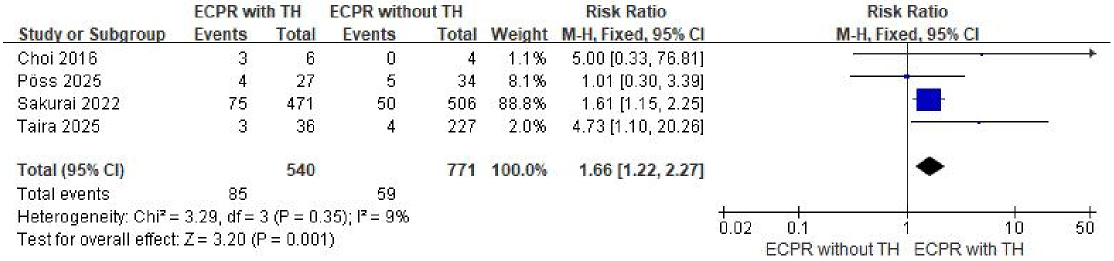

Pooled analysis using fixed-effects model. Diamond indicates overall effect size; vertical dashed line indicates null effect (RR = 1); horizontal lines indicate 95% confidence intervals for individual studies; square size reflects study weight. TH: therapeutic hypothermia; ECPR: extracorporeal cardiopulmonary resuscitation.

Supplemental Figure 32 Forest plot of meta-analysis for favorable neurological outcome at 3 months. (Only observational studies were pooled)

	ECPR with TH		ECPR without TH			Risk Ratio
Study or Subgroup	Events	Total	Events	Total	Weight	M-H, Fixed, 95% CI
Kagawa 2015	12	48	3	39	61.0%	3.25 [0.99, 10.71]
Kim 2014	5	14	3	38	29.8%	4.52 [1.24, 16.49]
Maekawa 2013	8	26	0	26	9.2%	17.00 [1.03, 280.07]
**Total (95% CI)**		**88**		**103**	**100.0%**	**4.90 [2.10, 11.44]**
Total events	25		6			

Heterogeneity: $Chi^2 = 1.23$, df = 2 (P = 0.54); $I^2 = 0\%$
Test for overall effect: Z = 3.67 (P = 0.0002)

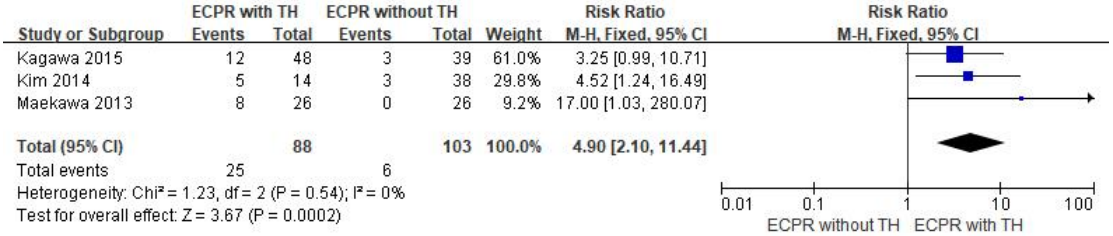

Pooled analysis using fixed-effects model. Diamond indicates overall effect size; vertical dashed line indicates null effect (RR = 1); horizontal lines indicate 95% confidence intervals for individual studies; square size reflects study weight. TH: therapeutic hypothermia; ECPR: extracorporeal cardiopulmonary resuscitation.

Supplemental Figure 33 Forest plot of meta-analysis for hemorrhage complications in ECPR patients treated with versus without therapeutic hypothermia.

(a) RCTs

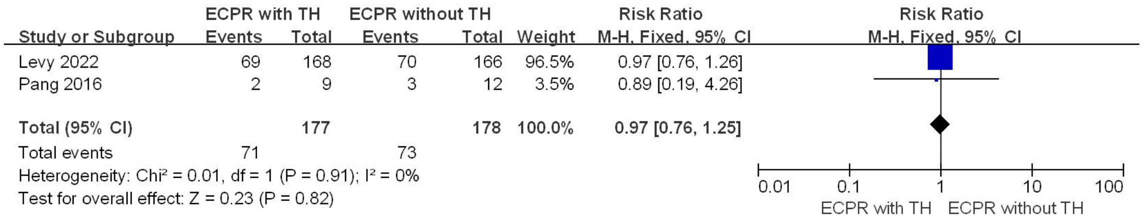

(b) observational studys

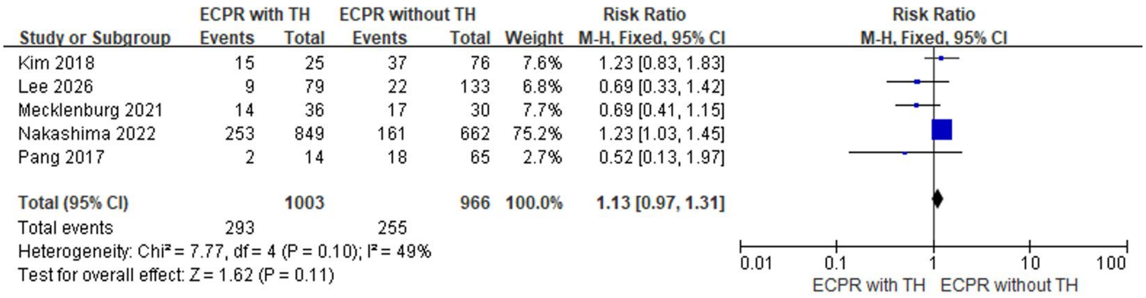

Pooled analysis using a fixed-effects model. Diamond indicates overall effect size; vertical dashed line indicates null effect (RR = 1); horizontal lines indicate 95% confidence intervals for individual studies; square size reflects study weight. TH: therapeutic hypothermia; ECPR: extracorporeal cardiopulmonary resuscitation. (a) represents the results of meta-analysis of RCT studies and (b) represents the results of meta-analysis of observational studies.

Supplemental Figure 34 Forest plot of meta-analysis for infection complications in ECPR patients treated with versus without therapeutic hypothermia.

(a) RCTs

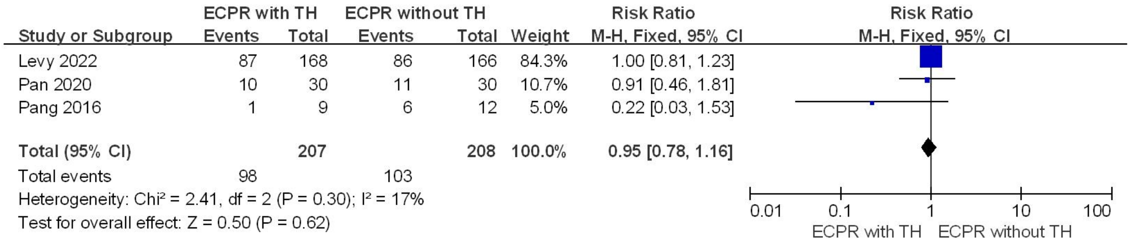

(b) observational studys

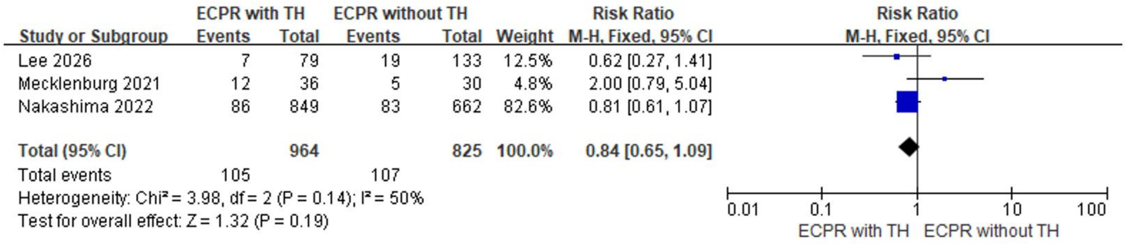

Pooled analysis using a fixed-effects model. Diamond indicates overall effect size; vertical dashed line indicates null effect (RR = 1); horizontal lines indicate 95% confidence intervals for individual studies; square size reflects study weight. TH: therapeutic hypothermia; ECPR: extracorporeal cardiopulmonary resuscitation. (a) represents the results of meta-analysis of RCT studies and (b) represents the results of meta-analysis of observational studies.

Supplemental Figure 35 Forest plot of meta-analysis for limb ischaemia complications in ECPR patients treated with versus without therapeutic hypothermia.

(a) RCTs

	ECPR with TH		ECPR without TH			Risk Ratio
Study or Subgroup	Events	Total	Events	Total	Weight	M-H, Fixed, 95% CI
Pan 2020	9	30	7	30	67.1%	1.29 [0.55, 3.00]
Pang 2016	2	9	4	12	32.9%	0.67 [0.15, 2.87]
**Total (95% CI)**		**39**		**42**	**100.0%**	**1.08 [0.52, 2.24]**
Total events	11		11			

Heterogeneity: Chi² = 0.58, df = 1 (P = 0.45); I² = 0%
Test for overall effect: Z = 0.21 (P = 0.83)

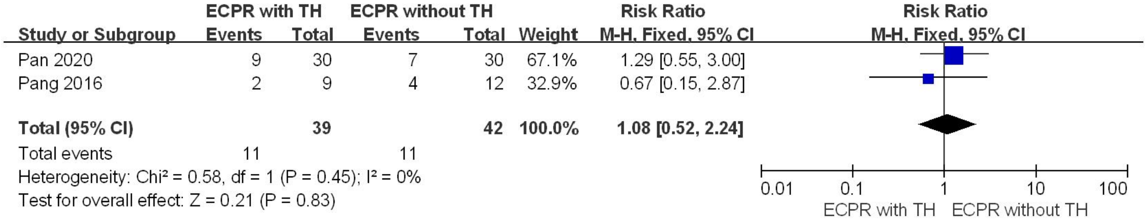

(b) observational studys

	ECPR with TH		ECPR without TH			Risk Ratio
Study or Subgroup	Events	Total	Events	Total	Weight	M-H, Fixed, 95% CI
Kim 2018	3	25	7	76	5.8%	1.30 [0.36, 4.66]
Nakashima 2022	60	849	46	662	86.0%	1.02 [0.70, 1.47]
Pang 2017	2	14	14	65	8.3%	0.66 [0.17, 2.60]
**Total (95% CI)**		**888**		**803**	**100.0%**	**1.00 [0.71, 1.42]**
Total events	65		67			

Heterogeneity: Chi² = 0.52, df = 2 (P = 0.77); I² = 0%
Test for overall effect: Z = 0.02 (P = 0.98)

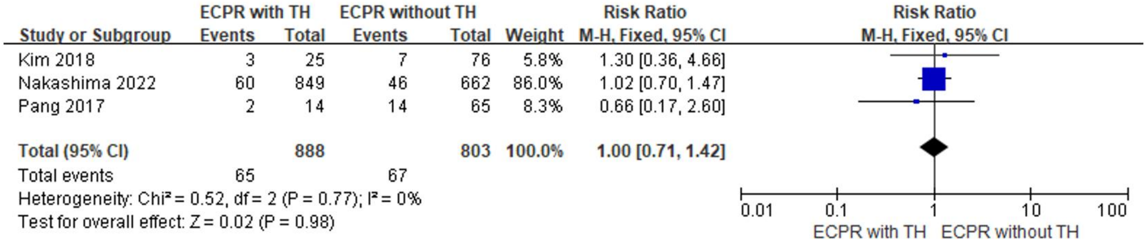

Pooled analysis using a fixed-effects model. Diamond indicates overall effect size; vertical dashed line indicates null effect (RR = 1); horizontal lines indicate 95% confidence intervals for individual studies; square size reflects study weight. TH: therapeutic hypothermia; ECPR: extracorporeal cardiopulmonary resuscitation. (a) represents the results of meta-analysis of RCT studies and (b) represents the results of meta-analysis of observational studies.

Supplemental Figure 36 Forest plot of meta-analysis for acute kidney injury complications in ECPR patients treated with versus without therapeutic hypothermia.

(a) RCTs

Study or Subgroup	ECPR with TH Events	ECPR with TH Total	ECPR without TH Events	ECPR without TH Total	Weight	Risk Ratio M-H, Fixed, 95% CI
Pan 2020	15	30	14	30	59.8%	1.07 [0.63, 1.81]
Pang 2016	8	9	11	12	40.2%	0.97 [0.73, 1.29]
**Total (95% CI)**		**39**		**42**	**100.0%**	**1.03 [0.73, 1.45]**
Total events	23		25			

Heterogeneity: Chi² = 0.19, df = 1 (P = 0.66); I² = 0%

Test for overall effect: Z = 0.17 (P = 0.86)

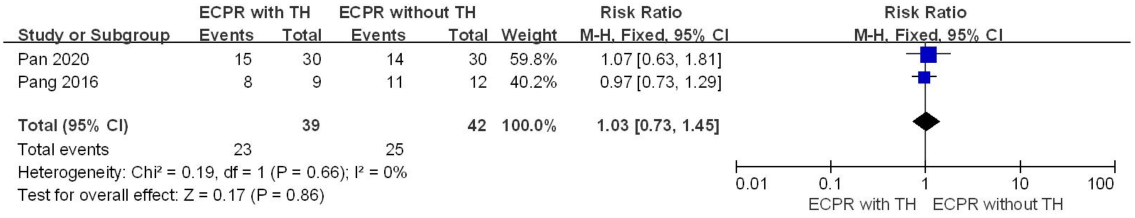

(b) observational studys

Study or Subgroup	ECPR with TH Events	ECPR with TH Total	ECPR without TH Events	ECPR without TH Total	Weight	Risk Ratio M-H, Fixed, 95% CI
Mecklenburg 2021	19	36	10	30	40.6%	1.58 [0.88, 2.86]
Pang 2017	10	14	45	65	59.4%	1.03 [0.71, 1.49]
**Total (95% CI)**		**50**		**95**	**100.0%**	**1.26 [0.90, 1.76]**
Total events	29		55			

Heterogeneity: Chi² = 1.68, df = 1 (P = 0.20); I² = 40%

Test for overall effect: Z = 1.33 (P = 0.18)

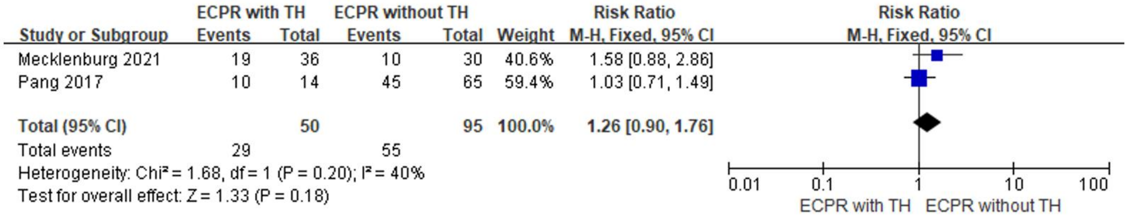

Pooled analysis using a fixed-effects model. Diamond indicates overall effect size; vertical dashed line indicates null effect (RR = 1); horizontal lines indicate 95% confidence intervals for individual studies; square size reflects study weight. TH: therapeutic hypothermia; ECPR: extracorporeal cardiopulmonary resuscitation. (a) represents the results of meta-analysis of RCT studies and (b) represents the results of meta-analysis of observational studies.

Supplemental Figure 37 Forest plot of meta-analysis for liver injury complications in ECPR patients treated with versus without therapeutic hypothermia.(Only observational studies were pooled)

	ECPR with TH		ECPR without TH			Risk Ratio
Study or Subgroup	Events	Total	Events	Total	Weight	M-H, Fixed, 95% CI
Mecklenburg 2021	27	36	27	30	81.4%	0.83 [0.67, 1.04]
Pang 2017	3	14	19	65	18.6%	0.73 [0.25, 2.14]
Total (95% CI)		50		95	100.0%	0.81 [0.63, 1.06]
Total events	30		46			
Heterogeneity: Chi² = 0.08, df = 1 (P = 0.78); I² = 0%						
Test for overall effect: Z = 1.52 (P = 0.13)						

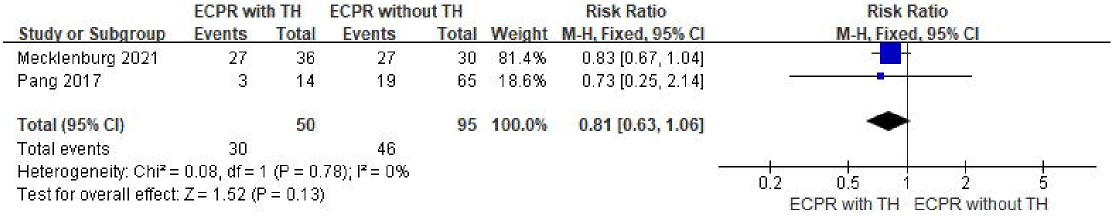

Pooled analysis using a fixed-effects model. Diamond indicates overall effect size; vertical dashed line indicates null effect (RR = 1); horizontal lines indicate 95% confidence intervals for individual studies; square size reflects study weight. TH: therapeutic hypothermia; ECPR: extracorporeal cardiopulmonary resuscitation.

Sensitivity analysis: leave-one-out method

Supplemental Figure 38 Forest plot of the sensitivity analysis for favorable neurological outcome at discharge (by excluding the Ijuin 2025 study).(Only observational studies were pooled)

Study or Subgroup	Experimental Events	Experimental Total	Control Events	Control Total	Weight	Risk Ratio M-H, Random, 95% CI
Al-Kawaz 2022	3	10	8	29	3.9%	1.09 [0.36, 3.32]
Dennis 2017	7	15	6	22	5.9%	1.71 [0.72, 4.09]
Han 2019	8	26	6	74	5.0%	3.79 [1.45, 9.91]
Ijuin 2025	43	293	37	180	0.0%	0.71 [0.48, 1.06]
Kim 2018	8	25	26	76	9.2%	0.94 [0.49, 1.79]
Kim 2024	11	69	10	69	6.9%	1.10 [0.50, 2.42]
Kobata 2020	5	18	1	4	1.5%	1.11 [0.17, 7.09]
Lee 2026	23	79	14	133	10.2%	2.77 [1.51, 5.06]
Otani 2020	11	33	13	123	8.2%	3.15 [1.56, 6.38]
Pang 2017	6	14	10	65	6.3%	2.79 [1.21, 6.40]
Ryu 2017	14	25	9	17	11.0%	1.06 [0.60, 1.87]
Ryu 2019	16	36	62	238	15.5%	1.71 [1.12, 2.61]
Sakamoto 2014	30	231	2	29	2.6%	1.88 [0.47, 7.47]
Shih 2024	12	55	3	30	3.5%	2.18 [0.67, 7.13]
Yamada 2021	38	202	8	66	8.1%	1.55 [0.76, 3.16]
Yukawa 2017	9	50	2	29	2.4%	2.61 [0.60, 11.26]
**Total (95% CI)**		**888**		**1004**	**100.0%**	**1.74 [1.38, 2.20]**
Total events	201		180			

Heterogeneity: Tau² = 0.04; Chi² = 18.02, df = 14 (P = 0.21); I² = 22%

Test for overall effect: Z = 4.67 (P < 0.00001)

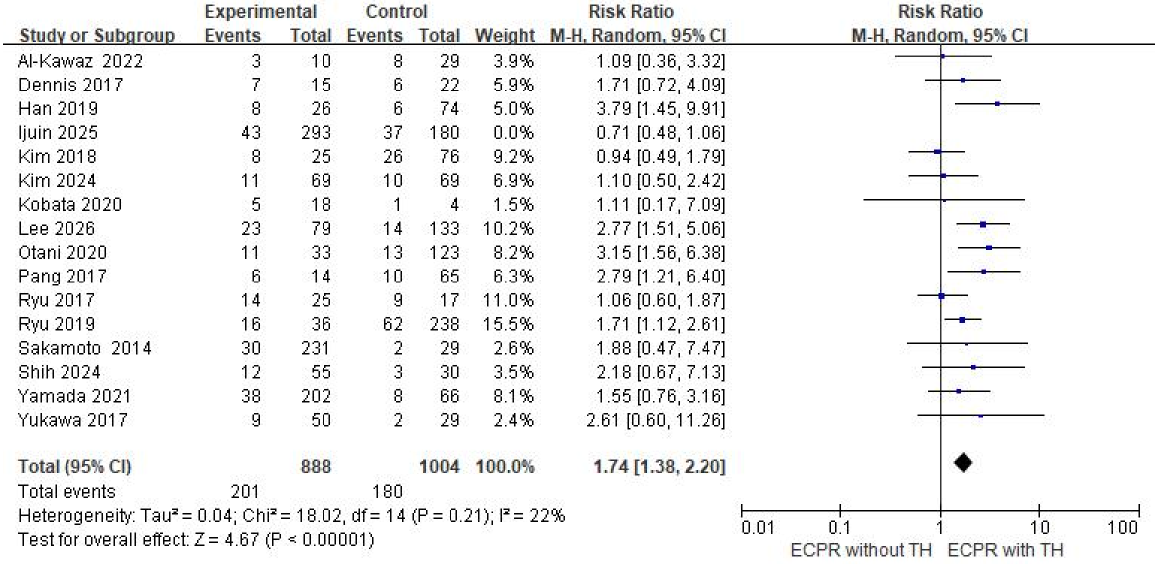

Supplemental Figure 39 Forest plot of the sensitivity analysis for survival at 1 month (by excluding the Lee 2016 study).(Only observational studies were pooled)

	ECPR with TH		ECPR without TH			Risk Ratio
Study or Subgroup	Events	Total	Events	Total	Weight	M-H, Random, 95% CI
Choi 2016	3	6	0	4	4.3%	5.00 [0.33, 76.81]
Kim 2021	10	38	26	146	44.8%	1.48 [0.78, 2.79]
Lee 2016	6	18	4	5	0.0%	0.42 [0.19, 0.92]
Mecklenburg 2021	14	36	14	30	50.9%	0.83 [0.48, 1.46]
**Total (95% CI)**		**80**		**180**	**100.0%**	**1.16 [0.65, 2.08]**
Total events	27		40			

Heterogeneity: Tau² = 0.09; Chi² = 3.05, df = 2 (P = 0.22); I² = 34%
Test for overall effect: Z = 0.51 (P = 0.61)

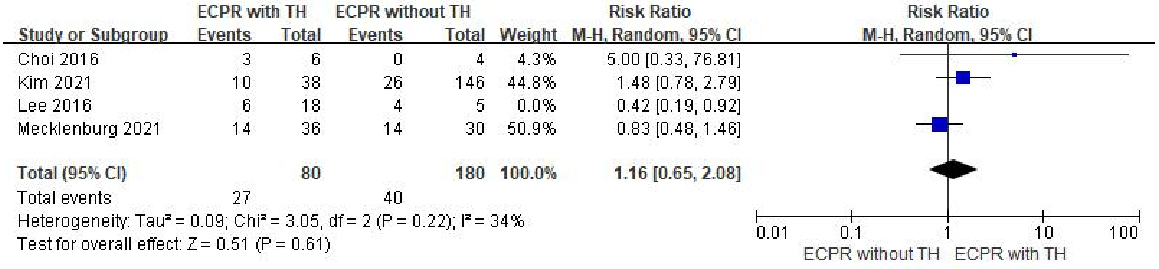

Supplemental Figure 40 Forest plot of the sensitivity analysis for hemorrhage (by excluding the Mecklenburg 2021 study).(Only observational studies were pooled)

Study or Subgroup	ECPR with TH Events	ECPR with TH Total	ECPR without TH Events	ECPR without TH Total	Weight	Risk Ratio M-H, Random, 95% CI
Kim 2018	15	25	37	76	25.3%	1.23 [0.83, 1.83]
Lee 2026	9	79	22	133	9.3%	0.69 [0.33, 1.42]
Mecklenburg 2021	14	36	17	30	0.0%	0.69 [0.41, 1.15]
Nakashima 2022	253	849	161	662	62.6%	1.23 [1.03, 1.45]
Pang 2017	2	14	18	65	2.9%	0.52 [0.13, 1.97]
**Total (95% CI)**		**967**		**936**	**100.0%**	**1.13 [0.90, 1.43]**
Total events	279		238			

Heterogeneity: Tau² = 0.01; Chi² = 3.86, df = 3 (P = 0.28); I² = 22%

Test for overall effect: Z = 1.07 (P = 0.29)

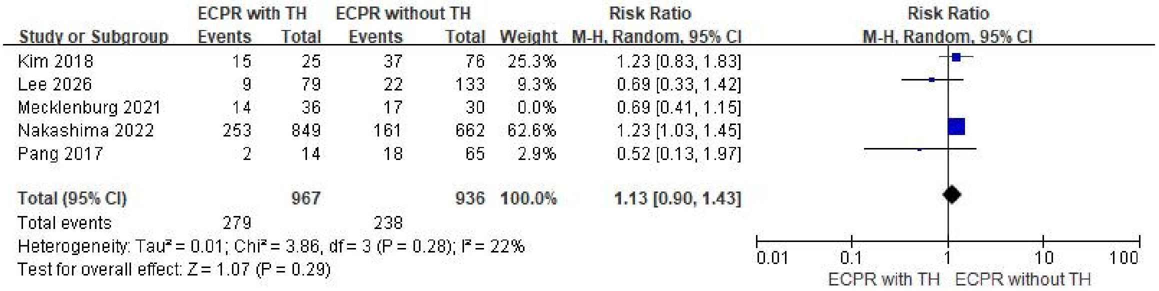

Supplemental Figure 41 Forest plot of the sensitivity analysis for infection (by excluding the Mecklenburg 2021 study).(Only observational studies were pooled)

Study or Subgroup	ECPR with TH Events	ECPR with TH Total	ECPR without TH Events	ECPR without TH Total	Weight	Risk Ratio M-H, Random, 95% CI
Lee 2026	7	79	19	133	10.7%	0.62 [0.27, 1.41]
Mecklenburg 2021	12	36	5	30	0.0%	2.00 [0.79, 5.04]
Nakashima 2022	86	849	83	662	89.3%	0.81 [0.61, 1.07]
**Total (95% CI)**		**928**		**795**	**100.0%**	**0.79 [0.60, 1.03]**
Total events	93		102			

Heterogeneity: Tau² = 0.00; Chi² = 0.36, df = 1 (P = 0.55); I² = 0%
Test for overall effect: Z = 1.76 (P = 0.08)

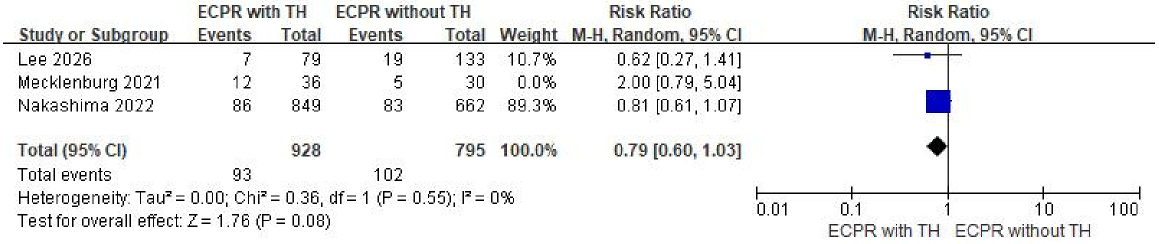

Supplemental Figure 42 Forest plot of the sensitivity analysis for favorable neurological outcome at discharge (by excluding the Ijuin 2025 study and Kim 2018 study).(Only observational studies were pooled)

Study or Subgroup	Experimental Events	Experimental Total	Control Events	Control Total	Weight	Risk Ratio M-H, Random, 95% CI
Al-Kawaz 2022	3	10	8	29	3.8%	1.09 [0.36, 3.32]
Dennis 2017	7	15	6	22	6.0%	1.71 [0.72, 4.09]
Han 2019	8	26	6	74	5.0%	3.79 [1.45, 9.91]
Ijuin 2025	43	293	37	180	0.0%	0.71 [0.48, 1.06]
Kim 2018	8	25	26	76	0.0%	0.94 [0.49, 1.79]
Kim 2024	11	69	10	69	7.2%	1.10 [0.50, 2.42]
Kobata 2020	5	18	1	4	1.4%	1.11 [0.17, 7.09]
Lee 2026	23	79	14	133	11.5%	2.77 [1.51, 5.06]
Otani 2020	11	33	13	123	8.8%	3.15 [1.56, 6.38]
Pang 2017	6	14	10	65	6.5%	2.79 [1.21, 6.40]
Ryu 2017	14	25	9	17	12.8%	1.06 [0.60, 1.87]
Ryu 2019	16	36	62	238	20.3%	1.71 [1.12, 2.61]
Sakamoto 2014	30	231	2	29	2.5%	1.88 [0.47, 7.47]
Shih 2024	12	55	3	30	3.3%	2.18 [0.67, 7.13]
Yamada 2021	38	202	8	66	8.7%	1.55 [0.76, 3.16]
Yukawa 2017	9	50	2	29	2.2%	2.61 [0.60, 11.26]
**Total (95% CI)**		**863**		**928**	**100.0%**	**1.84 [1.48, 2.30]**
Total events	193		154			

Heterogeneity: Tau² = 0.02; Chi² = 14.29, df = 13 (P = 0.35); I² = 9%

Test for overall effect: Z = 5.42 (P < 0.00001)

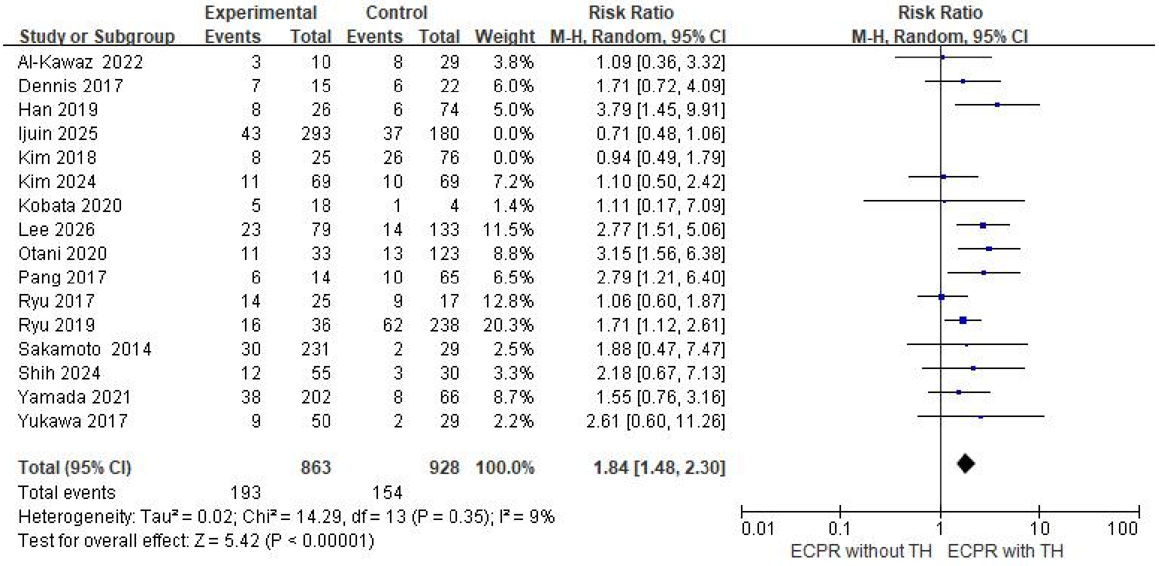

Supplemental Figure 43 Forest plot of the sensitivity analysis for survival at 1 month (by excluding the Lee 2016 study and Mecklenburg 2021 study).(Only observational studies were pooled)

	ECPR with TH		ECPR without TH			Risk Ratio
Study or Subgroup	Events	Total	Events	Total	Weight	M-H, Random, 95% CI
Choi 2016	3	6	0	4	5.1%	5.00 [0.33, 76.81]
Kim 2021	10	38	26	146	94.9%	1.48 [0.78, 2.79]
Lee 2016	6	18	4	5	0.0%	0.42 [0.19, 0.92]
Mecklenburg 2021	14	36	14	30	0.0%	0.83 [0.48, 1.46]
**Total (95% CI)**		**44**		**150**	**100.0%**	**1.57 [0.85, 2.92]**
Total events	13		26			

Heterogeneity: Tau² = 0.00; Chi² = 0.75, df = 1 (P = 0.39); I² = 0%
Test for overall effect: Z = 1.43 (P = 0.15)

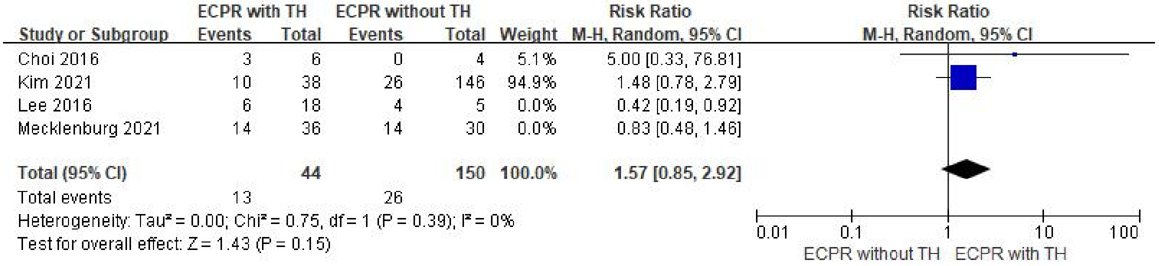

Publication Bias

Supplemental Figure 44 Contour-enhanced funnel plot for the assessment of publication bias in the meta-analysis of survival to discharge.(Only observational studies)

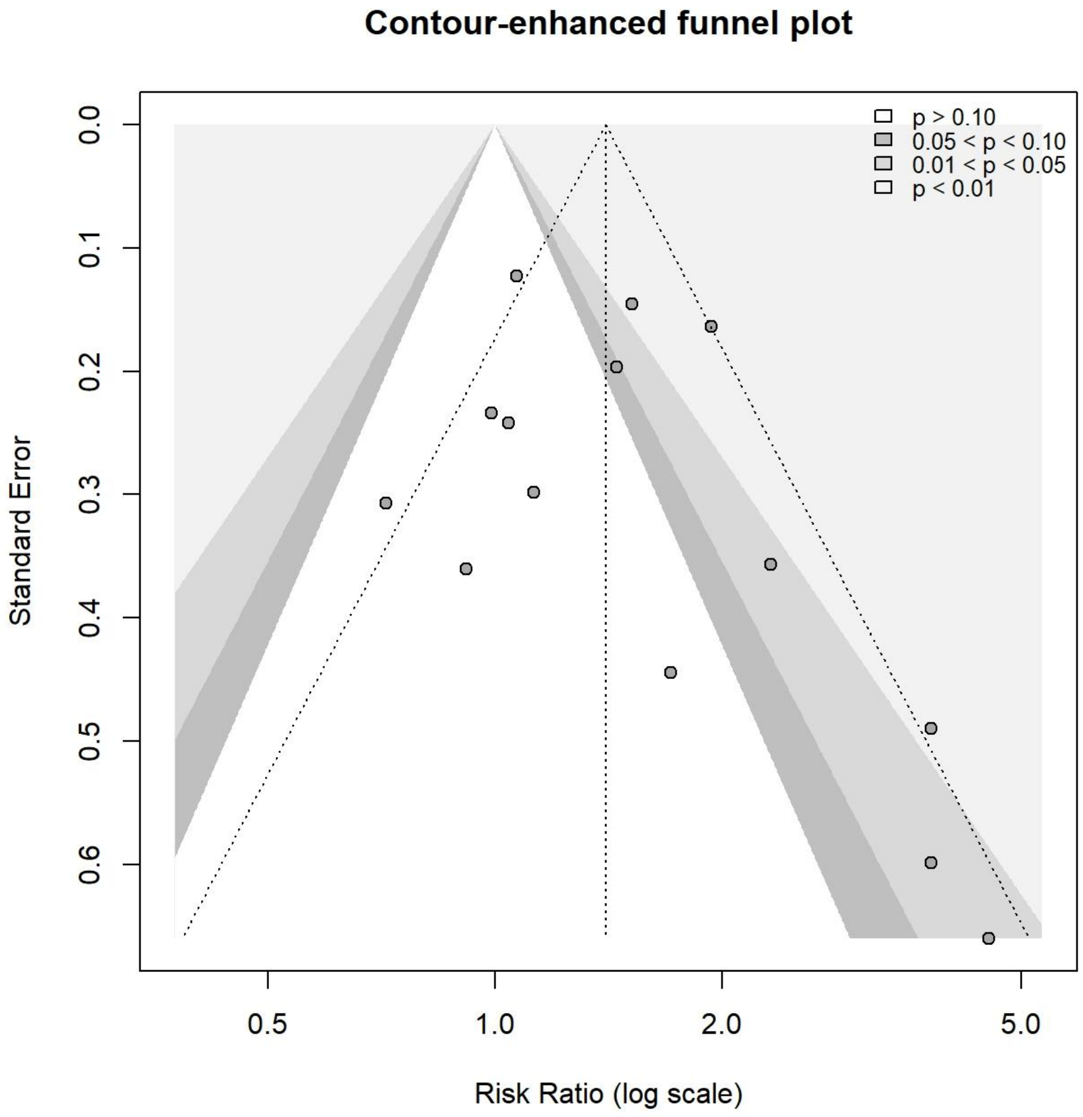

Supplemental Figure 45 Contour-enhanced funnel plot for the assessment of publication bias in the meta-analysis of neurological outcome at discharge.(Only observational studies)

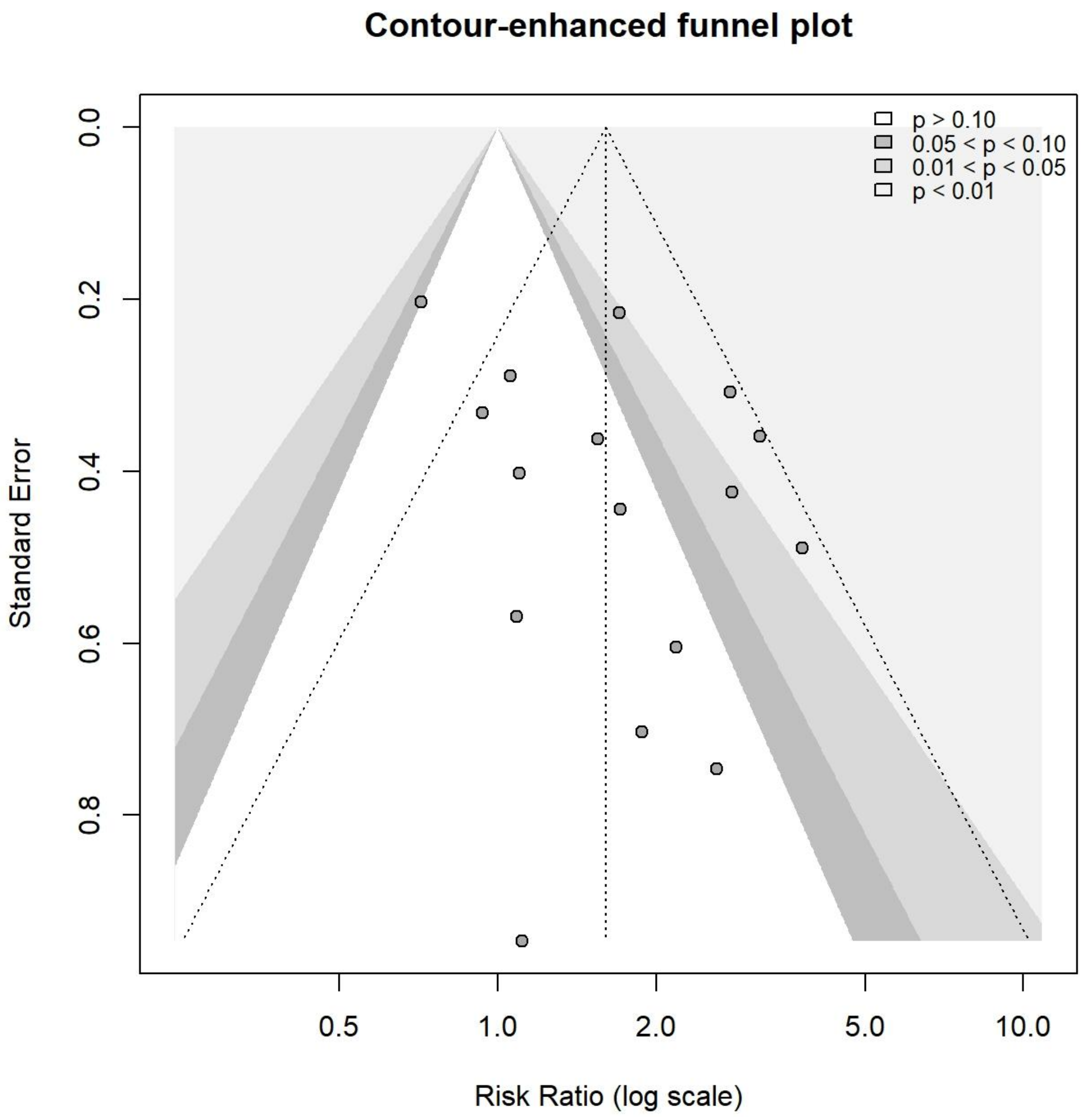

Certainty of Evidence

Supplemental Table 1 Summary of the quality of evidence.

Certainty assessment						Effect	Certainty
Outcome indicators	Risk of bias	Inconsistency	Indirectness	Imprecision	Other considerations	RR （95%CI）	
Survival at discharge,(RCT)	very serious[a]	not serious	not serious	very serious[b]	none	RR1.30 (0.70,2.40)	⊕◯◯◯ Very low a,b
Survival at discharge,(Non-RCT)	very serious[a]	serious[c]	not serious	not serious	none	RR 1.41 (1.14,1.74)	⊕◯◯◯ Very low a,c
Favorable neurological outcome at hospital discharge,(RCT)	very serious[a]	not serious	not serious	very serious[b]	none	RR1.80 (0.86,3.77)	⊕◯◯◯ Very low a,b
Favorable neurological outcome at hospital discharge(Non-RCT)	very serious[a]	serious[c]	not serious	not serious	none	RR 1.61 (1.21,2.14)	⊕◯◯◯ Very low a,b
Survival at 1 month,(Non-RCT)	very serious[a]	serious[c]	not serious	very serious[b]	none	RR 0.91 (0.46,1.82)	⊕◯◯◯ Very low a,b,c

Survival at 3 months,(Non-RCT)	very serious[a]	not serious	not serious	very serious[b]	none	RR 0.95 (0.73,1.24)	⊕◯◯◯ Very low a,b
Survival at 6 months,(RCT)	very serious[a]	not serious	not serious	very serious[b]	none	RR 1.37 (0.56,3.35)	⊕◯◯◯Very low a,b
Favorable neurological outcome at 1 month,(Non-RCT)	very serious[a]	not serious	not serious	serious[b]	none	RR 1.71 (1.12,2.64)	⊕◯◯◯ Very low a,b
Favorable neurological outcome at 3 months,(Non-RCT)	serious[a]	not serious	not serious	serious[b]	none	RR 4.33 (1.87,9.99)	⊕◯◯◯ Very low a,b
Hemorrhage,(RCT)	serious[a]	not serious	not serious	serious[b]	none	RR0.97 (0.76,1.25)	⊕⊕◯◯Low a,b
Hemorrhage,(Non-RCT)	serious[a]	serious[c]	not serious	not serious	none	RR 1.13 (0.97,1.35)	⊕◯◯◯ Very lowa,c
Infection,(RCT)	serious[a]	not serious	not serious	serious[b]	none	RR 0.95 (0.78,1.16)	⊕⊕◯◯Low a,b
Infection,(Non-RCT)	serious[a]	serious[c]	not serious	not serious	none	RR 0.84 (0.65,1.09)	⊕◯◯◯ Very lowa,c
Limb ischemia,(RCT)	not serious	not serious	not serious	very serious[b]	none	RR 1.08 (0.52,2.24)	⊕⊕◯◯Low b
Limb ischemia,(Non-RCT)	serious[a]	not serious	not serious	not serious	none	RR 1.00(0.71,1.42)	⊕◯◯◯ Very lowa

Acute kidney injury,(RCT)	not serious	not serious	not serious	very serious[b]	none	RR 1.03(0.73,1.45)	⊕⊕◯◯Low b
Acute kidney injury,(Non-RCT)	serious[a]	not serious	not serious	serious[b]	nonr	RR 1.26(0.90,1.76)	⊕◯◯◯ Very lowa,b
Liver injury,(Non-RCT)	serious[a]	not serious	not serious	serious[b]	none	RR 0.81 (0.67, 1.03)	⊕◯◯◯ Very lowa,b

CI: confidence interval; MD: mean difference; RR: risk ratio

Explanations

a. The main contribution of the pooled effect size came from studies with a high risk of bias

b. The sample size was small or/and the confidence interval was wide

c. There was moderate to high heterogeneity

Appendix A: Search strategy

PubMed

Search	Query	Results
#1	("heart arrest"[MeSH Terms] OR "cardiac arrest"[Title/Abstract] OR "out-of-hospital cardiac arrest"[MeSH Terms] OR "in-hospital cardiac arrest"[Title/Abstract] OR asystole[Title/Abstract])	87,272
#2	("extracorporeal membrane oxygenation"[MeSH Terms] OR "extracorporeal cardiopulmonary resuscitation"[Title/Abstract] OR "extracorporeal life support"[Title/Abstract] OR "mechanical circulation assistance"[Title/Abstract] OR ECMO[Title/Abstract] OR ECPR[Title/Abstract] OR ECLS[Title/Abstract] OR "cardiopulmonary resuscitation"[MeSH Terms] OR "extracorporeal circulation"[Title/Abstract] OR "oxygenators, membrane"[MeSH Terms] OR "life support, extracorporeal"[Title/Abstract])	60,024
#3	("hypothermia, induced"[MeSH Terms] OR "targeted temperature management"[Title/Abstract] OR "therapeutic hypothermia"[Title/Abstract] OR "moderate hypothermia"[Title/Abstract] OR "mild hypothermia*"[Title/Abstract] OR cryotherapy[Title/Abstract] OR "cold therapy"[Title/Abstract] OR TTM[Title/Abstract] OR rewarming[Title/Abstract])	42,453
#4	#1 AND #2 AND #3	1,760

Web of science(WOS)

Query	Results
(TS=('heart arrest' OR 'cardiac arrest' OR 'out-of-hospital cardiac arrest' OR 'in-hospital cardiac arrest' OR asystole))<br>AND<br>(TS=('extracorporeal membrane oxygenation' OR 'extracorporeal cardiopulmonary resuscitation' OR 'extracorporeal life support' OR 'mechanical circulation assistance' OR ECMO OR ECPR OR ECLS OR 'cardiopulmonary resuscitation' OR 'extracorporeal circulation' OR 'oxygenators, membrane' OR 'life support, extracorporeal'))<br>AND<br>(TS=('induced hypothermia' OR 'targeted temperature management' OR 'therapeutic hypothermia' OR 'moderate hypothermia' OR 'mild hypothermia*' OR cryotherapy OR 'cold therapy' OR TTM OR rewarming))	**5,369 results** from Web of Science Core Collection, Grants Index, Inspec®, KCI-Korean Journal Database, MEDLINE® , Policy Citation Index, ProQuest ™ Dissertations & Theses Citation Index, SciELO Citation Index.

Embase

Search	Query	Results
#1	'heart arrest'/exp OR 'heart arrest'	163,792
#2	'heart arrest':ab,ti,kw OR 'cardiac arrest':ab,ti,kw OR 'out-of-hospital cardiac arrest':ab,ti,kw OR 'in-hospital cardiac arrest':ab,ti,kw OR 'asystole':ab,ti,kw	95,588
#3	#1 OR #2	176,603
#4	'extracorporeal membrane oxygenation'/exp	60,085
#5	'extracorporeal membrane oxygenation':ab,ti,kw OR 'extracorporeal cardiopulmonary resuscitation':ab,ti,kw OR 'extracorporeal life support':ab,ti,kw OR 'mechanical circulation assistance':ab,ti,kw OR 'ECMO':ab,ti,kw OR 'ECPR':ab,ti,kw OR 'ECLS':ab,ti,kw OR 'cardiopulmonary resuscitation':ab,ti,kw OR 'extracorporeal circulation':ab,ti,kw OR 'membrane oxygenator'/exp OR 'life support, extracorporeal':ab,ti,kw	93,544
#6	#4 OR #5	112,885
#7	'induced hypothermia'/exp	21,640
#8	'induced hypothermia':ab,ti,kw OR 'targeted temperature management':ab,ti,kw OR 'therapeutic hypothermia':ab,ti,kw OR 'moderate hypothermia':ab,ti,kw OR 'mild hypothermia*':ab,ti,kw OR 'cryotherapy':ab,ti,kw OR 'cold therapy':ab,ti,kw OR 'TTM':ab,ti,kw OR 'rewarming':ab,ti,kw	43,358
#9	#7 OR #8	53,434
#10	#3 AND #6 AND #9	2,279

Cochrane library

Search	Query	Results
#1	([mh "Heart Arrest"] OR "heart arrest":ti,ab,kw OR "cardiac arrest":ti,ab,kw OR [mh "Out-of-Hospital Cardiac Arrest"] OR "in-hospital cardiac arrest":ti,ab,kw OR asystole:ti,ab,kw)	8,018
#2	([mh "Extracorporeal Membrane Oxygenation"] OR "extracorporeal membrane oxygenation":ti,ab,kw OR "extracorporeal cardiopulmonary resuscitation":ti,ab,kw OR "extracorporeal life support":ti,ab,kw OR ECMO:ti,ab,kw OR ECPR:ti,ab,kw OR ECLS:ti,ab,kw)	1,731
#3	([mh "Hypothermia, Induced"] OR "targeted temperature management":ti,ab,kw OR "therapeutic hypothermia":ti,ab,kw OR TTM:ti,ab,kw OR cryotherapy:ti,ab,kw OR "cold therapy":ti,ab,kw OR rewarming:ti,ab,kw)	6,431
#4	#1 AND #2 AND #3	14

Ovid

Search	Query	Results
1	exp heart arrest/	60,973
2	(heart arrest or cardiac arrest).mp.	75,539
3	exp out-of-hospital cardiac arrest/ or "out-of-hospital cardiac arrest".mp.	13,265
4	("in-hospital cardiac arrest" or asystole).mp.	6,523
5	1 or 2 or 3 or 4	94,087
6	exp extracorporeal membrane oxygenation/	18,356
7	exp cardiopulmonary resuscitation/	24,912
8	exp membrane oxygenator/	1,856
9	(extracorporeal membrane oxygenation or "extracorporeal cardiopulmonary resuscitation" or "extracorporeal life support").mp.	29,008
10	(ECMO or ECPR or ECLS).mp.	19,681
11	("mechanical circulation assistance" or cardiopulmonary resuscitation or "extracorporeal circulation" or "membrane oxygenator").mp.	53,391
12	6 or 7 or 8 or 9 or 10 or 11	81,663
13	exp hypothermia, induced/	22,893
14	("induced hypothermia" or "targeted temperature management" or "therapeutic hypothermia").mp.	9,084
15	("moderate hypothermia" or "mild hypothermia*" or cryotherapy or "cold therapy").mp.	16,990
16	(TTM or rewarming).mp.	8,476
17	13 or 14 or 15 or 16	47,361
18	5 and 12 and 17	2,299

CNKI

Query	Results
(SU=('心脏骤停' OR '呼吸心跳骤停' OR '心搏骤停' OR '心跳骤停' OR '心脏停搏' OR '猝死' OR 'SCA' OR 'CA') OR AB=('心脏骤停' OR '呼吸心跳骤停' OR '心搏骤停' OR '心跳骤停' OR '心脏停搏' OR '猝死' OR 'SCA' OR 'CA')) AND (SU=('体外生命支持' OR '体外心肺复苏' OR 'ECPR' OR 'ECMO' OR '体外膜肺氧合' OR '机械循环辅助') OR AB=('体外生命支持' OR '体外心肺复苏' OR 'ECPR' OR 'ECMO' OR '体外膜肺氧合' OR '机械循环辅助')) AND (SU=('目标体温管理' OR '亚体温' OR '低温' OR '治疗性低体温' OR '保护性低体温' OR '体温控制' OR '体温监测' OR '复温' OR '冷疗') OR AB=('目标体温管理' OR '亚体温' OR '低温' OR '治疗性低体温' OR '保护性低体温' OR '体温控制' OR '体温监测' OR '复温' OR '冷疗'))	113

Wanfang

Query	Results
(题名或关键词:("心脏骤停" OR "呼吸心跳骤停" OR "心搏骤停" OR "心跳骤停" OR "心脏停搏" OR "猝死" OR "SCA" OR "CA")) AND (题名或关键词:("体外膜肺氧合" OR "机械循环辅助" OR "体外生命支持" OR "体外心肺复苏" OR "ECPR" OR "ECMO")) AND (题名或关键词:("目标体温管理" OR "亚体温" OR "低温" OR "治疗性低体温" OR "保护性低体温" OR "体温控制" OR "体温监测" OR "复温" OR "冷疗"))	23

China Biology Medicine Disc(CBM)

Search	Query	Results
#1	("心脏骤停"[全部字段:智能] OR "呼吸心跳骤停"[全部字段:智能] OR "心搏骤停"[全部字段:智能] OR "心跳骤停"[全部字段:智能] OR "心脏停搏"[全部字段:智能] OR "猝死"[全部字段:智能] OR "SCA"[全部字段:智能] OR "CA"[全部字段:智能])	4273
#2	("体外膜肺氧合"[全部字段:智能] OR "机械循环辅助"[全部字段:智能] OR "体外生命支持"[全部字段:智能] OR "体外心肺复苏"[全部字段:智能] OR "ECPR"[全部字段:智能] OR "ECMO"[全部字段:智能])	2102
#3	("目标体温管理"[全部字段:智能] OR "亚体温"[全部字段:智能] OR "低温"[全部字段:智能] OR "治疗性低体温"[全部字段:智能] OR "保护性低体温"[全部字段:智能] OR "体温控制"[全部字段:智能] OR "体温监测"[全部字段:智能] OR "复温"[全部字段:智能] OR "冷疗"[全部字段:智能])	3893
#4	#1 AND #2 AND #3	45

Appendix B: PRISMA Checklist

Section/topic	#	Checklist item	Reported on page #
**TITLE**			
Title	1	Identify the report as a systematic review, meta-analysis, or both.	1
**ABSTRACT**			
Structured summary	2	Provide a structured summary including, as applicable: background; objectives; data sources; study eligibility criteria, participants, and interventions; study appraisal and synthesis methods; results; limitations; conclusions and implications of key findings; systematic review registration number.	1,2
**INTRODUCTION**			
Rationale	3	Describe the rationale for the review in the context of what is already known.	2,3
Objectives	4	Provide an explicit statement of questions being addressed with reference to participants, interventions, comparisons, outcomes, and study design (PICOS).	4
**METHODS**			
Protocol and registration	5	Indicate if a review protocol exists, if and where it can be accessed (e.g., Web address), and, if available, provide registration information including registration number.	3
Eligibility criteria	6	Specify study characteristics (e.g., PICOS, length of follow-up) and report characteristics (e.g., years considered, language, publication status) used as criteria for eligibility, giving rationale.	4
Information sources	7	Describe all information sources (e.g., databases with dates of coverage, contact with study authors to identify additional studies) in the search and date last searched.	3,4
Search	8	Present full electronic search strategy for at least one database, including any limits used, such that it could be repeated.	3,4
Study selection	9	State the process for selecting studies (i.e., screening, eligibility, included in systematic review, and, if applicable, included in the meta-analysis).	4,5& Figure 1
Data collection process	10	Describe method of data extraction from reports (e.g., piloted forms, independently, in duplicate) and any processes for obtaining and confirming data from investigators.	5
Data items	11	List and define all variables for which data were sought (e.g.,	5

		PICOS, funding sources) and any assumptions and simplifications made.	
Risk of bias in individual studies	12	Describe methods used for assessing risk of bias of individual studies (including specification of whether this was done at the study or outcome level), and how this information is to be used in any data synthesis.	5,6
Summary measures	13	State the principal summary measures (e.g., risk ratio, difference in means).	6
Synthesis of results	14	Describe the methods of handling data and combining results of studies, if done, including measures of consistency (e.g., I^2) for each meta-analysis.	6
Risk of bias across studies	15	Specify any assessment of risk of bias that may affect the cumulative evidence (e.g., publication bias, selective reporting within studies).	5,6
Additional analyses	16	Describe methods of additional analyses (e.g., sensitivity or subgroup analyses, meta-regression), if done, indicating which were pre-specified.	6
**RESULTS**			
Study selection	17	Give numbers of studies screened, assessed for eligibility, and included in the review, with reasons for exclusions at each stage, ideally with a flow diagram.	6 & Figure 1
Study characteristics	18	For each study, present characteristics for which data were extracted (e.g., study size, PICOS, follow-up period) and provide the citations.	7 & Table 1
Risk of bias within studies	19	Present data on risk of bias of each study and, if available, any outcome level assessment (see item 12).	7 & Figure 2,3
Results of individual studies	20	For all outcomes considered (benefits or harms), present, for each study: (a) simple summary data for each intervention group (b) effect estimates and confidence intervals, ideally with a forest plot.	7,8
Synthesis of results	21	Present results of each meta-analysis done, including confidence intervals and measures of consistency.	Figure 4,5 &Table 2, 3

Risk of bias across studies	22	Present results of any assessment of risk of bias across studies (see Item 15).	9
Additional analysis	23	Give results of additional analyses, if done (e.g., sensitivity or subgroup analyses, meta-regression [see Item 16]).	8
**DISCUSSION**			
Summary of evidence	24	Summarize the main findings including the strength of evidence for each main outcome; consider their relevance to key groups (e.g., healthcare providers, users, and policy makers).	9
Limitations	25	Discuss limitations at study and outcome level (e.g., risk of bias), and at review-level (e.g., incomplete retrieval of identified research, reporting bias).	11
Conclusions	26	Provide a general interpretation of the results in the context of other evidence, and implications for future research.	11,12
**FUNDING**			
Funding	27	Describe sources of funding for the systematic review and other support (e.g., supply of data); role of funders for the systematic review.	12

From: Moher D, Liberati A, Tetzlaff J, Altman DG, The PRISMA Group (2009). Preferred Reporting Items for Systematic Reviews and Meta-Analyses: The PRISMA Statement. PLoS Med 6(7): e1000097. doi:10.1371/journal.pmed1000097
